# The “Hidden” Reductive [2+2+1]‐Cycloaddition Chemistry of 2‐Phosphaethynolate Revealed by Reduction of a Th‐OCP Linkage

**DOI:** 10.1002/anie.202012506

**Published:** 2020-12-22

**Authors:** Jingzhen Du, Gábor Balázs, Ashley J. Wooles, Manfred Scheer, Stephen T. Liddle

**Affiliations:** ^1^ Department of Chemistry The University of Manchester Oxford Road Manchester M13 9PL UK; ^2^ Institute of Inorganic Chemistry University of Regensburg Universitätsstr. 31 93053 Regensburg Germany

**Keywords:** 2-phosphaethynolate, actinides, phosphorus heterocycles, reduction, thorium

## Abstract

The reduction chemistry of the newly emerging 2‐phosphaethynolate (OCP)^−^ is not well explored, and many unanswered questions remain about this ligand in this context. We report that reduction of [Th(Tren^TIPS^)(OCP)] (**2**, Tren^TIPS^=[N(CH_2_CH_2_NSiPr^i^_3_)]^3−^), with RbC_8_ via [2+2+1] cycloaddition, produces an unprecedented hexathorium complex [{Th(Tren^TIPS^)}_6_(μ‐OC_2_P_3_)_2_(μ‐OC_2_P_3_H)_2_Rb_4_] (**5**) featuring four five‐membered [C_2_P_3_] phosphorus heterocycles, which can be converted to a rare oxo complex [{Th(Tren^TIPS^)(μ‐ORb)}_2_] (**6**) and the known cyclometallated complex [Th{N(CH_2_CH_2_NSiPr^i^_3_)_2_(CH_2_CH_2_SiPr^i^_2_CHMeCH_2_)}] (**4**) by thermolysis; thereby, providing an unprecedented example of reductive cycloaddition reactivity in the chemistry of 2‐phosphaethynolate. This has permitted us to isolate intermediates that might normally remain unseen. We have debunked an erroneous assumption of a concerted fragmentation process for (OCP)^−^, rather than cycloaddition products that then decompose with [Th(Tren^TIPS^)O]^−^ essentially acting as a protecting then leaving group. In contrast, when KC_8_ or CsC_8_ were used the phosphinidiide C−H bond activation product [{Th(Tren^TIPS^)}Th{N(CH_2_CH_2_NSiPr^i^_3_)_2_[CH_2_CH_2_SiPr^i^_2_CH(Me)CH_2_C(O)μ‐P]}] (**3**) and the oxo complex [{Th(Tren^TIPS^)(μ‐OCs)}_2_] (**7**) were isolated.

## Graphical abstract

A reaction with the overall appearance of (OCP)^−^ concerted cleavage is revealed to proceed through a [2+2+1]‐cycloaddition reaction intermediate, introducing an unprecedented example of (OCP)^−^ cycloaddition under reducing conditions to the established neutral and oxidative cycloaddition classes of (OCP)^−^ reactivity.

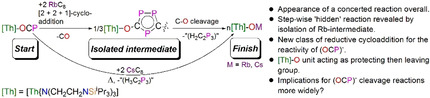

## Introduction

There is burgeoning interest in the emerging, fundamental chemistry of the 2‐phosphaethynolate (OCP)^−^ anion, not only because regular investigations of this anion became practicable only around a decade ago but also because it is a valence isoelectronic, *P*‐analogue of the ubiquitous cyanate (OCN)^−^ anion.^[1]^ In terms of the fundamental redox chemistry of (OCP)^−^, it has been found that this anion is readily oxidised, ostensibly to the heterobicylic dianion, (P_4_C_4_O_4_)^2−^, via radical coupling.^[2]^ Furthermore, cycloaddition reactions of (OCP)^−^ under oxidising or neutral conditions have become well established, producing a variety of novel three, four‐, five‐, or six‐membered phosphorus heterocycles.^[1]^ In contrast, the reduction chemistry of (OCP)^−^ is in its infancy,[^1^, ^3^] but the clear picture already is that this closed‐shell anion resists reduction, to avoid populating antibonding orbitals, inevitably leading, when reduction can be effected, to spontaneous fragmentation of the O‐C‐P unit in the majority of cases. Indeed, this has been observed in many low‐valent early d‐block and f‐block cases.[^1^, ^3^] Thus, there are no (OCP)^−^ cycloaddition reactions yet reported under reducing conditions.

Notwithstanding the proclivity of (OCP)^−^ to fragment on reduction, a few notable examples report divergent reaction outcomes. Reduction of [Sc{HC(CMeNAr)_2_}(OAr)(THF)}(OCP)] (Ar=2,6‐Pr^i^_2_C_6_H_3_) with KC_8_ generates a strongly activated transient radical dianion (OCP)^2−**⋅**^, which dimerises to give (OCPPCO)^4−^.^[4]^ In contrast, we reported that reduction of [U(Tren^TIPS^)(OCP)] (Tren^TIPS^={N(CH_2_CH_2_NSiPr^i^_3_)_3_}^3−^) with KC_8_/2.2.2‐cryptand results in trapping of the radical dianion [OCP]^2−**⋅**^, formally a product of reduction by U^III^, in the mixed‐valence diuranium(III/IV) complex [{U(Tren^TIPS^)}_2_(μ‐OCP)][K(2.2.2‐cryptand)] that features a strongly activated, highly bent O‐C‐P unit (126.6(9)°).^[5]^ Seemingly moderate activation of the O‐C‐P linkage was found when [Th(OCP){PhC(NSiMe_3_)_2_}_3_] was reacted with [Ni(COD)_2_] to afford [Th(μ‐OCP)Ni(COD)}{PhC(NSiMe_3_)_2_}_3_], which also exhibits a bent O‐C‐P moiety (148.1(3)°).^[6]^ Given these prior results, and since Th^III^ is known to be more reactive than U^III^,^[7]^ this raised the question of whether there would be different behaviour of activated (OCP)^−^ with a Th‐ rather than U‐Tren^TIPS^ system.

Here, we report the first example of a cycloaddition reaction of (OCP)^−^ under reducing conditions. Specifically, we find a [2+2+1] cycloaddition reaction of (OCP)^−^, which is likely mediated by a Th^III^ species that results from reduction of a stable Th^IV^‐OCP linkage by strong reductants MC_8_ (M=K, Rb, Cs). This produces an unprecedented hexathorium cluster containing four five‐membered [C_2_P_3_] phosphorus heterocycles. Thermolysis of this cluster produces the ultimate products of this reaction, a rare example of a thorium‐oxo species and a cyclometallated complex via O−C and Th−C bond cleavage. The isolation of the cluster intermediate, which might normally have remained unseen, as well as the final oxo product permits us to realise that a reaction that has the superficial overall appearance of a concerted cleavage of the O−C bond of the O‐C‐P unit is in fact far more complicated than previously thought. The sterically demanding [Th(Tren^TIPS^)O]^−^ unit has essentially performed the role of protecting then leaving group, allowing the presence of an otherwise “hidden” [2+2+1] cycloaddition reaction to be recognised. These observations, together with the isolation of phosphinidiide and C−H activation by‐products when M=K, suggest that reductive (OCP)^−^ fragmentation reactions more widely are likely more elaborate than previously thought.

## Results and Discussion

**Figure 1:**
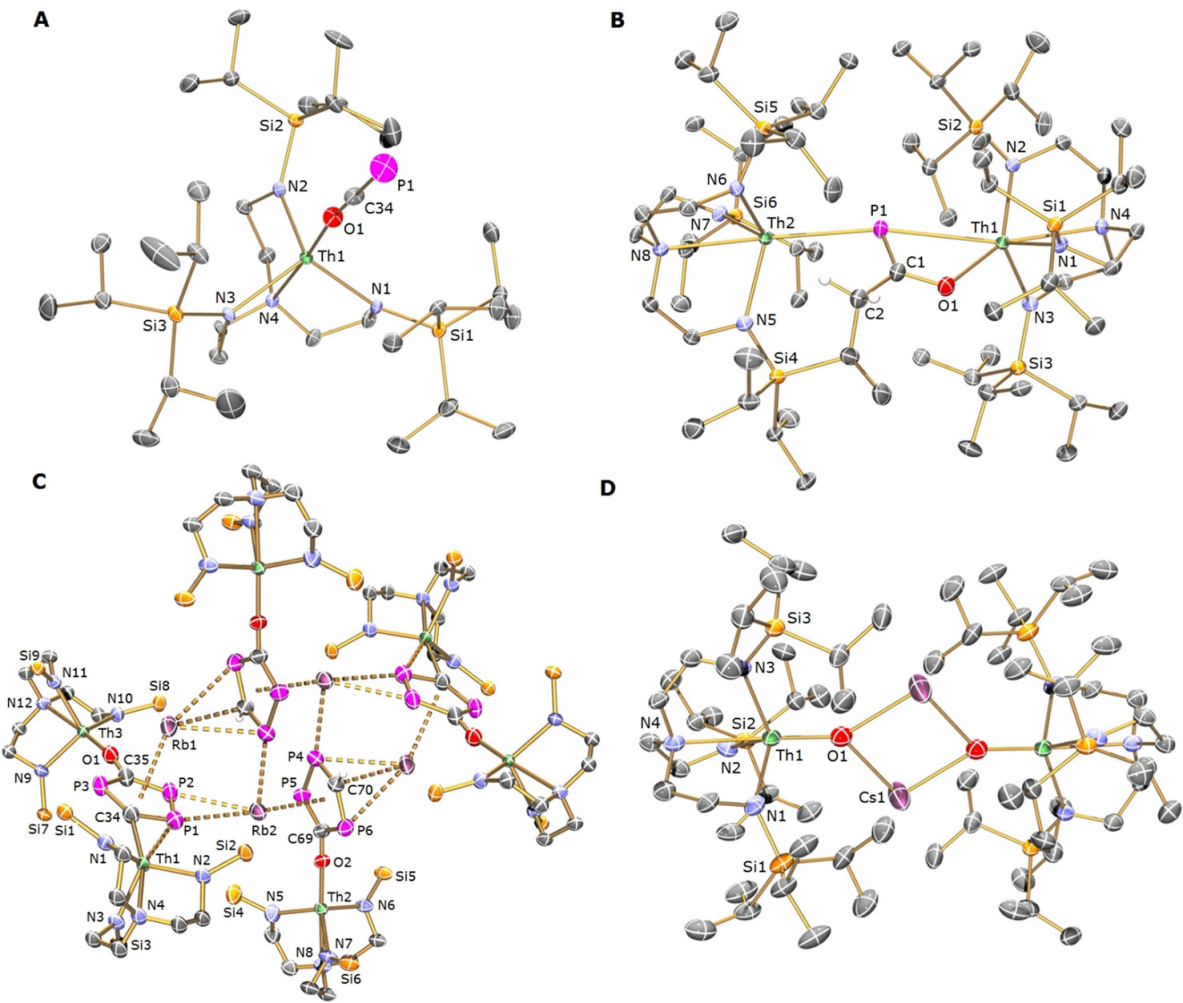
Molecular structures of A) **2**, B) **3**, C) **5**, and D) **7** at 150 K depicted with selective labels for the asymmetric components and 40 % probability displacement ellipsoids.^[25]^ Hydrogen atoms except for two hydrogen atoms at C2 in **3** (B) and two C_2_P_3_H atoms in **5** (C), isopropyl groups for **5** (C), minor disorder components, lattice solvent molecules, and any C−H⋅⋅⋅Rb interactions are omitted for clarity. The structure of **6** is very similar to **7** so it is not shown here.

**Scheme 1:**
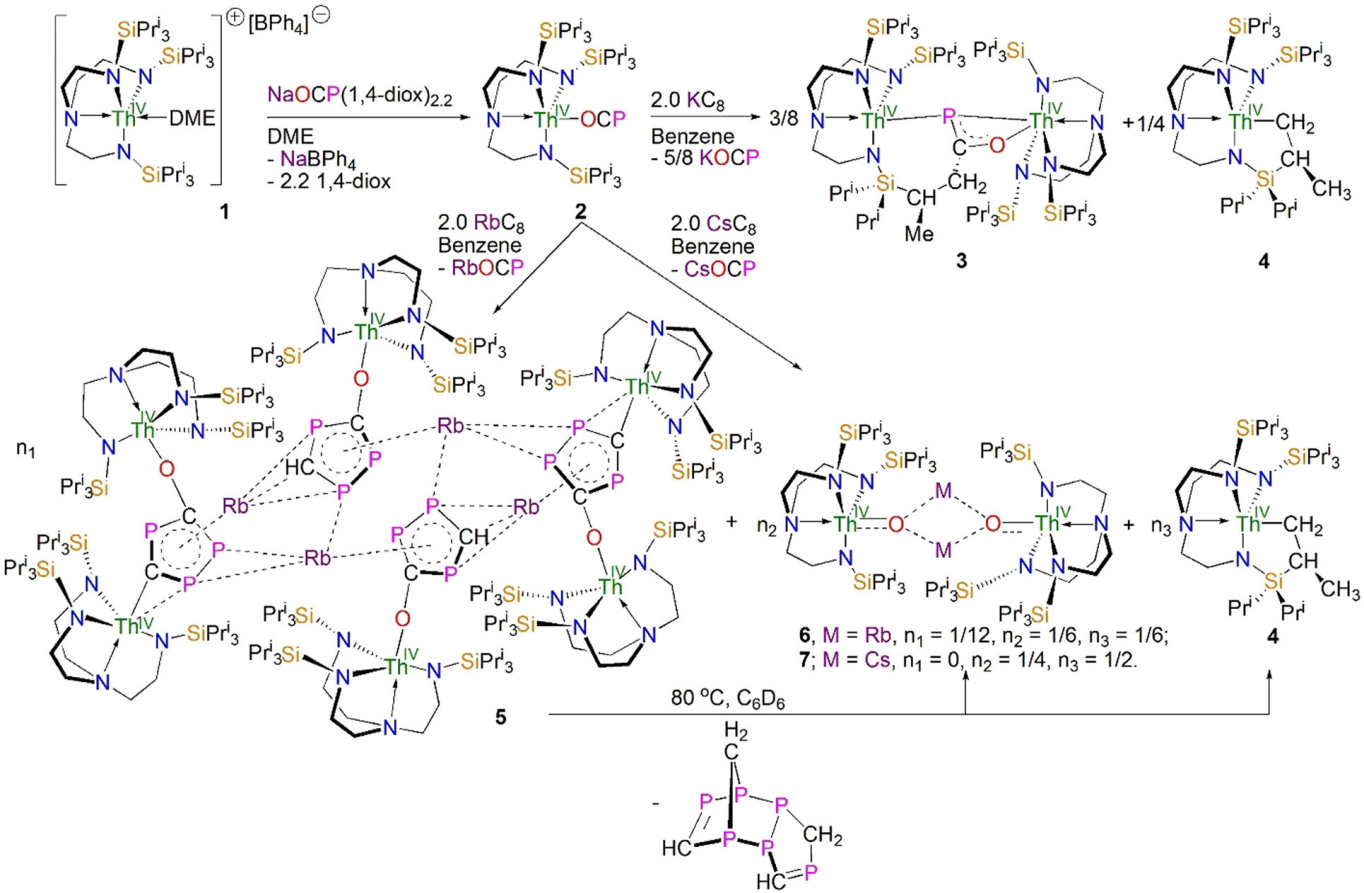
Synthesis of the thorium(IV)‐OCP complex **2**, and reduction of **2** to prepare **3** and **5**–**7** using MC_8_ (M=K, Rb, Cs) reagents. The proposed structure of the eliminated H_6_C_4_P_6_ from **5** is one of several possible isomers. However, if polymeric species do not form, it seems most likely on the basis of Ref. [24].

***Synthesis, characterisation, and solid‐state structure of 2***. Treatment of [Th(Tren^TIPS^)(DME)][BPh_4_]^[8]^ (**1**) with [Na(OCP)(1,4‐diox)_2.2_] in DME gives [Th(Tren^TIPS^)(OCP)] (**2**) in 52 % crystalline yield (Scheme 1).^[9]^ The ^1^H NMR spectrum of **2** exhibits three resonances spanning the range from 1.0 to 4.0 ppm, with the protons of the isopropyl groups being overlapped, which was previously observed in the closely related compound [Th(Tren^TIPS^)(N_3_)].^[10]^ The ^13^C{^1^H} NMR spectrum of **2** shows five resonances within the range 10.0 to 160.0 ppm, with the (OCP)^−^ carbon resonance identified at 157.6 ppm. Single resonances at 3.4 and −339.9 ppm in the ^29^Si{^1^H} and ^31^P{^1^H} NMR spectra of **2**, respectively, support the *C*_3*v*_ symmetric formulation (Supporting Information, Figures S7–S10). In the ATR‐IR spectrum of **2**, the antisymmetric stretch of (OCP)^−^ is clearly identified as a strong absorption at 1678 cm^−1^, but the symmetric stretch could not be assigned due to the complexity of the fingerprint region (Figure S2). The solid structure of **2** (Figure 1) reveals Th−O, O−C, and C−P distances of 2.334(3), 1.246(5), and 1.544(5) Å, and Th‐O‐C and O‐C‐P angles of 173.1(3) and 178.8(5)°, respectively, suggesting overall^[11]^ the dominance of P≡C‐O^−^, rather than ^−^P=C=O, resonance forms, as commonly observed when this anion is coordinated to electropositive metal ions.^[3]^ The Th−N_amide_ and Th−N_amine_ distances are unremarkable. These data compare well to other actinide‐OCP complexes.[^5^, ^6^, ^12^]

In order to ascertain the likely reducibility of **2**, we computed the DFT geometry optimised structure of **2** and its reduced Th^III^ form **2^−^**.^[9]^ Interestingly, the LUMO of **2** is computed to be mainly a 7s/5f hybrid with only minor (<10 %) 6d character and it has the appearance of a m_l_=3 orbital (ϕ‐type) with three of the six lobes diminished due to destructive interference from the 7s component (Figure S18). In **2^−^** (Figure S19), the HOMO is still Th‐centred, though now this orbital is roughly composed of equal 7s, 6d, and 5f contributions; the quasi‐degenerate LUMO and LUMO+1 of **2^−^**, which are π* orbitals of (OCP)^−^ lie ≈0.5 eV above the HOMO, and there is clear overlap of the Th‐coefficient with the O‐C‐P components, and so MLCT resulting in spin density transfer to the O‐C‐P unit with concomitant radical couplings is easily energetically feasible.

***Reduction chemistry of 2***. With **2** in‐hand, and a theoretical basis for its likely reduction chemistry established, we examined its reduction with MC_8_ (M=K, Rb, Cs).^[9]^ Unfortunately, the use of toluene as reaction solvent gives intractable products irrespective of the used alkali metal, but reactions in benzene gave identifiable products. Addition of benzene to a mixture of KC_8_ and **2** in a 2:1 ratio resulted in a dark yellow‐brown slurry, from which, after work‐up, a few colourless crystals of the phosphinidiide complex [{Th(Tren^TIPS^)} Th {N(CH_2_CH_2_NSi Pr^i^_3_)_2_ [CH_2_CH_2_Si Pr^i^_2_CH(Me)CH_2_C(O)μ‐P]}] (**3**) were isolated and structurally determined (Scheme 1). Unfortunately, the yield of **3** is intrinsically low and its formation is always accompanied by the known thorium cyclometallated compound [Th{N(CH_2_CH_2_NSiPr^i^_3_)_2_(CH_2_CH_2_SiPr^i^_2_CHMeCH_2_)}]^[13]^ (**4**) and other unidentified side‐products. The similar solubility of these products renders the isolation of **3** in pure form problematic, which hampered its further spectroscopic characterisation. However, by NMR spectroscopy **3** and **4** are produced in the ratio shown in Scheme 1. In contrast, using RbC_8_ in the reduction produced mixed products, including the unprecedented hexathorium complex [{Th(Tren^TIPS^)}_6_(μ‐OC_2_P_3_)_2_(μ‐OC_2_P_3_H)_2_Rb_4_] (**5**), and a rare bridging thorium oxo complex [{Th(Tren^TIPS^)(μ‐ORb)}_2_] (**6**). Although the yields for **5** and **6** are inherently low (16 and 8 %, respectively, based on thorium), they can be isolated in bulk scales and pure forms that allows further spectroscopic characterisation. When CsC_8_ is used, the caesium oxo congener [{Th(Tren^TIPS^)(μ‐OCs)}_2_] (**7**) is isolated from the reaction mixture in 28 % yield. In all these reactions, the formations of cyclometallated **4** and MOCP were always detected by ^1^H NMR and ATR‐IR spectroscopic analysis of the crude product mixtures,^[9]^ suggesting that **4** derives from a transient thorium(III) species [Th(Tren^TIPS^)] generated by reductive salt elimination. The different reaction outcomes as a function of alkali metal is interesting to note. It is well known^[14]^ that Group 1 metal‐arene interactions tend to become more favourable as the metal becomes larger, and it would seem that Rb is the optimal size match for the [C_2_P_3_] ring. It then follows that Cs is likely too large and reactive leading to destabilisation and the reaction pushing through to completion (oxo **7**) whereas for K it would seem an alternative reaction pathway occurs as perhaps K is too small.

***Solid‐state characterisation and spectroscopic data of 3 and 5**–**7***. In order to confirm the formulations of **3** and **5**–**7**, we determined their crystal structures (Figure 1; Figure S1). The molecular structure of **3** reveals a slightly bent Th1‐P1‐Th2 core (165.66(7)°), with Th1−P1 and Th2−P1 bond distances of 3.1890(17) and 3.0158(17) Å, respectively, which are longer than the single Th−P bond lengths in the related dithorium phosphinidiide complex [{Th(Tren^TIPS^)}_2_(μ‐PH)] (2.8982(17) and 2.8977(17) Å).^[8]^ The long Th1−O1 bond distance (2.365(5) Å), and short C1−O1 (1.309(9) Å) and C1−P1 (1.714(8) Å) bond lengths indicate the charge delocalisation within the [O1‐C1‐P1] unit, and the C1−C2 bond distance of 1.549(11) Å is typical of a C−C single bond.^[9]^

The salient feature of the solid‐state structure of **5** is that four five‐membered [C_2_P_3_] phosphorus heterocycles are bridged by four rubidium ions and six thorium centres, the latter either directly coordinated to the rings or via oxygen atoms. The structure has an inversion centre at the central point of the [Rb2‐P4‐Rb2A‐P4A] plane and the two different sets of [C_2_P_3_] rings are reminiscent of the phosphorus‐substituted cyclopentadienyl ring [(^*t*^BuC)_2_P_3_]^−^ which has been used to prepare a handful of ferrocene‐like transition metal complexes.^[15]^ A similar anionic analogue of [{(Me_3_SiO)C}_2_P_3_]^−^ was also reported from the reaction of Me_3_SiX (X=Cl, OTf, N_3_) with 3.0 equivalents of Na(OCP).^[16]^ The Th2−O2 and Th3−O1 bond distances of 2.119(16) and 2.200(13) Å, respectively, are significantly shorter than the sum of the covalent single bond radii for thorium and oxygen (2.38 Å),^[9]^ suggesting the O[C_2_P_3_] ligand is a strong donor, but they compare well with the Th−OMe bond length (2.1563 Å) for a related [Th(Tren^TIPS^)(OMe)] complex,^[10]^ as well as those for the reported Th‐OAr complexes.^[17]^ The coordination of Th1 to one of the [C_2_P_3_] rings is unique with a long Th1−C34 bond distance (2.579(18) Å), which compares well with Th−C single bonds for **4** (2.590(8) Å) and the benzyl compound [Th(Tren^TIPS^)(CH_2_Ph)] (2.563(4) Å) supported by the same Tren^TIPS^ ligand.^[13]^ A weak Th1−P1 interaction (3.210(9) Å) is also found. Notably, the centroid displacements of Rb to the corresponding [C_2_P_3_] rings (≈3.1 Å) and the short P−P (≈2.05 Å), P−C (≈1.75 Å) bond lengths within these phosphorus heterocycles are statistically invariant to each other and the O−C bonds are slightly contracted (≈1.30 Å), reflecting the negative charges delocalised within all these [C_2_P_3_] rings. This is also supported by the ^31^P{^1^H} NMR study of **5**, which exhibits 6 multiplets in the narrow range (215 to 265 ppm) with typical phosphorus coupling constants *J*_PP_=523.6 and 523.3 Hz, ^2^ *J*_PP_=10.7 and 63.1 Hz, respectively (Figure S13). The non‐decoupled ^31^P NMR spectrum shows that the three sets of phosphorus resonances in the higher chemical shift range from 255 to 262 ppm display further couplings patterns, but ^1^H‐^31^P coupling information could not be extracted (Figure S14). Nevertheless, this confirms the presence of two different types of [C_2_P_3_] rings in **5**, one with a proton [P4‐P5‐C69‐P6‐C70‐H] and one without [P1‐P2‐C35‐P3‐C34], which has been further ascertained by the multi‐nuclear NMR spectroscopic studies. For example, the ^1^H and ^29^Si{^1^H} NMR spectra of **5** show two pairs of CH_2_ (α, 3.69–3.78 ppm and β, 2.70–2.76 ppm, respectively) and silicon resonances (3.32 and 3.73 ppm), respectively (Figures S11 and S12), consistent with two Tren ligands coordinated to two different types of [C_2_P_3_] rings. In addition, the two‐dimensional (2D) ^1^H‐^13^C HSQC NMR spectrum (HSQC=Heteronuclear Single Quantum Correlation) suggests the proton resonances of CH for the [P4‐P5‐C69‐P6‐C70‐H] rings are overlapped with those of β‐CH_2_ for Tren ligands. Although the carbon resonances are weak in the 2D ^1^H‐^13^C HSQC NMR spectrum due to poor solubility of **5** in C_6_D_6_, the resonance around 65 ppm can be identified as the CH carbon resonance and the coupling interactions with the proton resonances around 2.7 ppm are clearly seen (Figure S15). The CH proton for [P4‐P5‐C69‐P6‐C70‐H] ring may reasonably come from the cyclometallate **4** because a proton would be released when **4** is formed and departs. The typical feature of the ATR‐IR spectrum of **5** (Figure S3) is the absence of the strong absorption at 1678 cm^−1^ compared with that for the precursor **2**, however, similar to the reported anionic analogue of [{(Me_3_SiO)C}_2_P_3_]^−^,^[16]^ the P‐C and P‐P vibrational modes for these [C_2_P_3_] rings could not be definitively identified in their ATR‐IR spectra and this might be due to their overlapping or being coupled with other stretching modes for the Tren^TIPS^ ligand.

Complexes **6** and **7** are isostructural to one another, and their molecular structures are similar to our reported diactinide parent imido complexes [{An(Tren^TIPS^)(μ‐NHM)}_2_] (An=Th, U; M=Li, Na, K, Rb, Cs)[^10^, ^18^] and diuranium nitrides [{U(Tren^TIPS^)(μ‐NM)}_2_].^[19]^ The Th−O bond distances in **6** (2.026(9) Å) and **7** (2.016(12) Å) are comparable to those for a few reported thorium oxo compounds, for example Th−O distances of 1.929(4) and 1.983(7) Å, were reported for [Th(O)(η^5^‐1,2,4‐(Me_3_C)_3_C_5_H_2_)_2_(DMAP)] (DMAP = 4‐dimethylaminopyridine)^[20]^ and [K(18‐crown‐6)][Th(O){N(SiMe_3_)_2_}_3_],^[21]^ respectively. The Th−N_amide_ and Th−N_amine_ distances in complexes **5**–**7** are unexceptional. Similar to the reported bridging imido and nitride complexes,[^10^, ^18^, ^19^] the poor solubility of **6** and **7** in aromatic solvent hampered their characterisation by NMR spectroscopy, but elemental analyses confirm their formulations. An analytical frequencies calculation on the crystallographic coordinates of **6** (full details of the electronic structures of **6** and **7** will be reported elsewhere) reveals principal Th=O stretches at ≈630 and ≈670 cm^−1^, and although the fingerprint regions of **6** and **7** are complex, as was found with other thorium‐oxos,[^20^, ^21^] strong bands at 625/669 and 628/667 cm^−1^ (Figures S4 and S5), respectively, are clearly discernible.

**Scheme 2:**
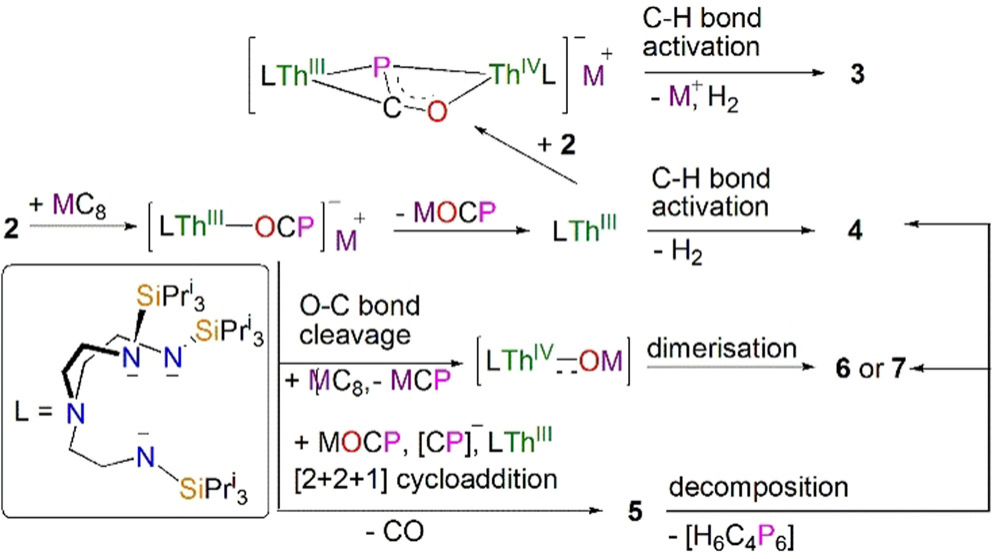
Proposed mechanisms for the formation of **3**–**7** from the reduction of **2**. The thorium ions in **2**–**7** are all thorium(IV). The proposed structure of the H_6_C_4_P_6_ by‐product is given in Scheme 1.

***Discussion of reduction chemistry of 2***. The identification of **3**–**7** from the reduction of **2** is in stark contrast to our prior reduction chemistry of uranium‐OCP system that only gave an isolable mixed‐valence diuranium(III/IV) complex.^[5]^ This likely reflects the more reactive, radical character of the O‐C‐P linkage promoted by low‐valent thorium(III) compared to uranium(III) (*cf*. the computed electronic structure of **2^−^**). The proposed reaction pathways are shown in Scheme 2.

That the cyclometallated species **4** is always produced in each reaction indicates that reductive salt elimination of MOCP derived from a putative thorium(III)‐OCP species [M][Th(Tren^TIPS^)(OCP)] commonly occurs to generate a highly unstable thorium(III) intermediate [Th(Tren^TIPS^)]. This latter species in turn likely then decomposes to **4** by activating the C−H bond of the methyl group with elimination of H_2_. This is in‐line with our previous findings that **4** and its uranium analogue not only result from acid‐base deprotonations,^[13]^ but also from low‐valent U^III^ [U(Tren^TIPS^)] slowly liberating H_2_ with concomitant oxidation to the U‐analogue of **4**;^[22]^ the corresponding thorium complex would be anticipated to be even more active in this regard.

Both the thorium(III) intermediates [M][Th(Tren^TIPS^)(OCP)] and [Th(Tren^TIPS^)] appear vitally important for producing **3** and **5**–**7**. For instance, [Th(Tren^TIPS^)] could further react with **2** to form a transient mixed‐valence dithorium(III/IV) complex [M][{Th(Tren^TIPS^)}_2_(μ‐OCP)], but unlike the stable and isolable uranium analogue,^[5]^ this species is clearly unstable, so would then undergo C−H bond activation to give **3**. Experimentally, we find that **4** does not react with **2**, ruling that out as a potential pathway to **3**. Either way, the ultimate phosphinidiide is notable. In contrast, the formation of **6** and **7** can be understood as overall fragmentation of the O‐C‐P unit by O−C bond cleavage mediated by a strongly reducing thorium(III) centre. Another similar strategy of reductive O−C bond cleavage to prepare actinide oxo species in the form of [K(18‐crown‐6)][An(O){N(SiMe_3_)_2_}_3_] (An=Th, U) has been reported.[^21^, ^23^] Nevertheless, complex **2** represents a new, useful precursor from which to access the thorium‐oxos **6** and **7**, which can potentially be converted to capped or terminal species.

In all of these products, the formation of intermediate **5** is certainly complex, and if it were not isolable and only **4**/**6**/**7** were observed then the overall O‐C cleavage reaction of the (OCP)^−^ unit would take on the specious appearance of a simple concerted cleavage reaction.[^1^, ^3^] Unfortunately, it is not realistic to determine the reaction profile computationally given the size of the system. However, the reaction can be rationalised as a typical [2+2+1] cycloaddition process involving production of [M][Th(Tren^TIPS^)(OCP)], which could dimerise with itself then eliminate **6**/**7** (giving an effective addition of (CP)^−^ overall, which would account for the additional equivalent of MC_8_ seemingly needed in these reactions to provide charge balance) and react with (OCP)^−^ with elimination of CO to give **5**. Alternatively, dimerised [M][Th(Tren^TIPS^)(OCP)] could react directly with (OCP)^−^ with concomitant elimination of CO and then **6**/**7**. Either way, the formation of the C_2_P_3_ ring occurs by a [2+2+1] cycloaddition reaction overall. Notably, **5** exhibits C_2_P_3_‐rings that are missing an oxo group, which can be accounted for by the formation of **6**/**7**, which with further MC_8_ could also produce the (CP)^−^ formally required in the formation of the C_2_P_3_ rings if (OCP)^−^ is not the source.

The multiple O−C and C−P bond cleavage, and P−C and P−P bond formation steps involved in the generation of [2+2+1] cycloaddition C_2_P_3_ rings is consistent with population of the O‐C‐P π* antibonding orbitals where the radical spin density is delocalised across the whole O‐C‐P unit, with all three atoms being activated, and this is in perfect agreement with the computed electronic structure of **2^−^**. Although examples of [2+2+1] cycloaddition have been proposed in group 14‐OCP systems to produce a similar [O_2_(C_2_P_3_)]^3−^,^[16]^ it has not yet been observed in actinide‐ or transition metal‐OCP chemistry nor in a reductive scenario. Importantly, thermolysis of **5** in C_6_D_6_ at 80 °C (Figure S17) gives **6** and **4**, with the presumed phosphorus‐dicyclopentadiene‐type species (H_6_C_4_P_6_) as the by‐product,^[24]^ confirming **5** as an intermediate to **6**. We suggest that intermediate **5** can be isolated at all and then converted to **6** in a tractable manner is due to the bulky [Th(Tren^TIPS^)O]^−^ unit acting as a protecting then leaving group to modulate the kinetics the of these reactions.

## Conclusion

To conclude, we have prepared the second example of a Th‐OCP complex **2** and examined its reduction chemistry using a series of strongly reducing alkali metal reagents MC_8_ (M=K, Rb, Cs). It was found that the isolable products from the reduction essentially depend on the identity of M involved. For the Rb case, the isolation of an unprecedented hexathorium species **5** from this reduction provides the first clear‐cut example of [2+2+1] cycloaddition in the reduction chemistry of 2‐phosphaethynolate. Indeed, this work introduces a new class of chemical reactivity of (OCP)^−^, adding cycloaddition under reductive conditions to oxidative and neutral classes of cycloaddition reactivities. Notably, thermolysis of **5** gives **4** and a rare thorium oxo species **6** revealing that [Th(Tren^TIPS^)(O)]^−^ can be a good protecting then leaving group, which is very rarely seen in actinide chemistry. This suggests that the [2+2+1] cycloaddition species **5** is an intermediate for the formation of thermal stable fragmentation products—a scenario that is normally hidden in OCP‐reduction chemistry. Thus, the complexity of what would otherwise be deemed a simple concerted cleavage reaction of (OCP)^−^ is revealed. This chemistry reflects the more reactive nature of low valent thorium species compared to uranium analogues and highlights that reductive (OCP)^−^ O‐ or P‐atom transfer fragmentation reactions in the wider context are likely to be more elaborate than previously imagined.

## Conflict of interest

The authors declare no conflict of interest.

## Supporting information

As a service to our authors and readers, this journal provides supporting information supplied by the authors. Such materials are peer reviewed and may be re‐organized for online delivery, but are not copy‐edited or typeset. Technical support issues arising from supporting information (other than missing files) should be addressed to the authors.

Supplementary
