# Supplementary material for: The “Hidden” Reductive [2+2+1]‐Cycloaddition Chemistry of 2‐Phosphaethynolate Revealed by Reduction of a Th‐OCP Linkage
Source: Angew Chem Int Ed Engl. 2020 Dec 22;60(3):1197–202. doi: 10.1002/anie.202012506 (PMC7839465; doi:10.1002/anie.202012506)
Supplement: Supplementary file 1 — Supplementary [file ANIE-60-1197-s001.pdf]

Angewandte Chemie
Eine Zeitschrift der Gesellschaft Deutscher Chemiker

Supporting Information

The "Hidden" Reductive [2+2+1]-Cycloaddition Chemistry of 2-Phosphaethynolate Revealed by Reduction of a Th-OCP Linkage

Jingzhen Du, Gábor Balázs, Ashley J. Wooles, Manfred Scheer, and Stephen T. Liddle**

anie_202012506_sm_miscellaneous_information.pdf

Experimental Details

General Procedures

All manipulations were carried out using Schlenk techniques, or an MBraun UniLab glovebox, under an atmosphere of dry nitrogen. Solvents were dried by passage through activated alumina towers and degassed before use. All solvents were stored over potassium mirrors except for ethers which were stored over activated 4 Å sieves. Deuterated solvent was distilled from potassium, degassed by three freeze-pump-thaw cycles and stored under dinitrogen. $[Th(Tren^{TIPS})(DME)][BPh_4]$ (**1**, $Tren^{TIPS} = \{N(CH_2CH_2NSi^iPr_3)_3\}^{3-}$) was prepared as described previously.[1] $Na(OCP)(dioxane)_{2.2}$ was prepared through modification of published procedures.[2,3] MC_8 (M = K, Rb, Cs) was prepared using literature methods.[4]

Single crystals were examined variously on either a) an Oxford Diffraction SuperNova Atlas CCD diffractometer using mirror-monochromated MoKα radiation (λ = 0.71073 Å), b) a Rigaku Xcalibur2 diffractometer equipped with an Atlas CCD area detector and a sealed tube source with graphite-monochromated MoKα radiation (λ = 0.71073 Å), or c) a Rigaku FR-X diffractometer equipped with a Hypix 6000HE photon counting pixel array detector with mirror-monochromated MoKα (λ = 0.71073 Å) or CuKα (λ = 1.5418 Å) radiation. Intensities were integrated from a sphere of data recorded on narrow (1.0°) frames by ω rotation. Cell parameters were refined from the observed positions of all strong reflections in each data set. Gaussian grid face-indexed absorption corrections with a beam profile correction were applied. The structures were solved either by dual methods using SHELXT[5] and all non-hydrogen atoms were refined by full-matrix least-squares on all unique F^2 values with anisotropic displacement parameters with exceptions noted in the respective cif files. Hydrogen atoms were refined with constrained geometries and riding thermal parameters; $U_{iso}(H)$ was set at 1.2 (1.5 for methyl groups) times U_{eq} of the parent atom. The largest

features in final difference syntheses were close to heavy atoms and were of no chemical significance. CrysAlisPro was used for control and integration,[6] and SHELXL and Olex2 were employed for structure refinement.[7,8] ORTEP-3 and POV-Ray were employed for molecular graphics.[9,10]

^{1}H, ^{13}C, ^{29}Si, and ^{31}P NMR spectra were recorded on a Bruker 400 spectrometer operating at 400, 101, 79, and 162 MHz, respectively; chemical shifts are quoted in ppm and are relative to TMS (^{1}H, ^{13}C, and ^{29}Si) and 85% H_3PO_4 (^{31}P). ATR-IR spectra were recorded on a Bruker Alpha spectrometer with a Platinum-ATR module in the glovebox. CHN microanalyses were carried out by Martin Jennings and Anne Davies at the University of Manchester.

Preparation of [Th(Tren^{TIPS})(OCP)] (2)

DME (30 ml) was added to a cold (–78 °C) mixture of **1** (2.50 g, 2.0 mmol) and NaOCP(dioxane)$_{2.2}$ (0.57 g, 2.2 mmol). The white slurry was allowed to warm to room temperature whilst stirring and stirred for further 24 h. Volatiles were removed *in vacuo* and the product was extracted into pentane (60 ml) and filtered. The filtrate was concentrated to approximate 5 mL, and then stored at –30 °C for two days to give colourless crystals of **2** suitable for a single-crystal X-ray diffraction study. After decanting the mother liquor, crystalline solid of **2** was dried *in vacuo*. Yield: 0.94 g, 52%. Anal. Calcd for $C_{34}H_{75}N_4OPSi_3Th$: C, 45.21; H, 8.37; N, 6.20%. Found: C, 45.46; H, 8.64; N, 6.00%. ^{1}H NMR (400 MHz, C_6D_6, 298 K): δ (ppm) 1.23 (br, 63H, C*H*(C*H*$_3$)$_2$), 2.58 (t, $^3J_{HH}$ = 4.8 Hz, 6H, C*H*$_2$CH$_2$), 3.52 (t, $^3J_{HH}$ = 4.8 Hz, 6H, CH$_2$C*H*$_2$). ^{13}C{^{1}H} NMR (101 MHz, C_6D_6, 298 K): δ (ppm) 12.49 (*C*H(CH$_3$)$_3$), 19.29 (CH(*C*H$_3$)$_3$), 46.84 (*C*H$_2$CH$_2$), 61.83 (CH$_2$*C*H$_2$), 157.60

(O*C*P). $^{29}Si\{^1H\}$ NMR (79 MHz, C_6D_6, 298 K): δ (ppm) 3.41. $^{31}P\{^1H\}$ NMR (162 MHz, C_6D_6, 298 K): δ (ppm) –339.91. FTIR *v*/cm^{-1}: 2939 (br, m), 2861 (br, m), 2845 (br, m), 1678 (vs, OCP^-), 1461 (m), 1382 (w), 1344 (w), 1285 (w), 1268 (w), 1134 (w), 1054 (m), 1007 (w), 920 (s), 881 (m), 859 (m), 817 (m), 743 (vs), 673 (s), 632 (s), 571 (m), 544 (m), 518 (m), 487 (m), 420 (w).

Preparation of [{Th(Tren^{TIPS})}Th{N(CH$_2$CH$_2$NSiPr$^i_3$)$_2$[CH$_2$CH$_2$SiPr$^i_2$CH(Me)CH$_2$C(O)μ-P]}] (3) and isolation of [Th{N(CH$_2$CH$_2$NSiPr$^i_3$)$_2$(CH$_2$CH$_2$SiPr$^i_2$CHMeCH$_2$)}] (4)

Benzene (40 ml) was added slowly to a pre-cooled (–78 °C) mixture of **2** (0.90 g, 1.00 mmol) and KC_8 (0.27 g, 2.00 mmol). The frozen mixture was left to thaw to room temperature and stirred for 24 h, during which time the formation of black graphite was observed, and the mixture turned into a yellow-brown slurry. The mixture was then filtered to give a yellow-brown solution. Removal of the volatiles afforded a dark red-brown oily residue. ^1H NMR study of the crude product indicates the formation of the known thorium cyclometallate complex **4**[1] as the main product, along with other unknown species. The oily residue was extracted with pentane (3 ml) and filtered. Storing the filtrate at −30 °C for 2 days gave a few colourless crystals of **3** suitable for a single-crystal X-ray diffraction study. However, the yield of **3** is inherently low, and the formation of **3** is always mixed with **4** and other unknown products. All these compounds have very similar solubility in the commonly used solvents, which renders the isolation of **3** in pure form impossible, impeding further spectroscopic characterisation.

Preparation of [{Th(Tren^{TIPS})}$_6$(μ-OC$_2$P$_3$)$_2$(μ-OC$_2$P$_3$H)$_2$Rb$_4$] (5) and conversion of 5 into [{Th(Tren^{TIPS})(μ-ORb)}$_2$] (6)

Benzene (40 ml) was added slowly to a pre-cooled (–78 °C) mixture of **2** (0.90 g, 1.00 mmol) and RbC_8 (0.36 g, 2.00 mmol). The frozen mixture was left to thaw to room temperature and stirred for 2 days, during which time the formation of black graphite was observed, and the mixture turned into a red slurry. The mixture was then filtered to give a red solution. Removal of the volatiles afforded a red-oily residue. ^{1}H NMR study of the crude product indicates the formation of **4** and [2+2+1] cycloaddition to give the hexathorium complex **5** (See Figure S16). Pentane (3 ml) was added to the residue, forming a red slurry with yellow solid **5**. The yellow solid was collected by filtration and washed with pentane (2 x 2 ml), and dried *in vacuo*. Yield: 0.16 g, 16% (based on thorium). Dark yellow crystals suitable for a single-crystal X-ray diffraction study were obtained either by storing a saturated solution in benzene or the red washings in pentane at room temperature for 3 days. Anal. Calcd for $C_{206}H_{452}N_{24}O_4P_{12}Rb_4Si_{18}Th_6$: C, 41.64; H, 7.67; N, 5.66%. Found: C, 42.14; H, 8.04; N, 5.62%. ^{1}H NMR (400 MHz, C_6D_6, 298 K): δ (ppm) 1.31-1.36 (m, 324H, CH(CH_3)$_2$), 1.47-1.58 (m, 56H, CH(CH$_3$)$_2$ and HC$_2$P$_3$), 2.70-2.76(m, 36H, CH_2CH$_2$), 3.69-3.78 (m, 38H, CH and CH$_2$CH_2). ^{29}Si{^{1}H} NMR (79 MHz, C_6D_6, 298 K): δ (ppm) 3.32 and 3.73. ^{31}P{^{1}H} NMR (162 MHz, C_6D_6, 298 K): δ (ppm) 261.14 (d, $^{1}J_{PP}$ = 523.61 Hz, $^{2}J_{PP}$ = 10.68 Hz), 257.91 (d, $^{1}J_{PP}$ = 523.61 Hz, $^{2}J_{PP}$ = 10.68), 255.80 (d, $^{2}J_{PP}$ = 10.6 Hz), 255.41 (d, $^{2}J_{PP}$ = 10.6 Hz), 221.22 (d, $^{1}J_{PP}$ = 523.32 Hz, $^{2}J_{PP}$ = 63.12 Hz), 217.99 (d, $^{1}J_{PP}$ = 523.61 Hz, $^{2}J_{PP}$ = 63.12 Hz). ATR-IR ν/cm^{-1}: 2938 (br, m), 2860 (br, m), 1460 (m), 1381 (w), 1274 (w), 1137 (m), 1057 (m), 1011 (w), 928 (s), 880 (s), 810 (w), 736 (vs), 670 (s), 627 (s), 599 (m), 545 (m), 514 (m), 441 (w). The ^{13}C{^{1}H} NMR spectrum was not obtained due to the compound is only partly soluble in aromatic solvents (benzene or toluene), and decomposes in polar solvent like THF. ATR-IR spectrum of the insoluble mixture with the graphite suggests the presence of RbOCP and

another complex which is insoluble in benzene at room temperature (See Figure S6, this compound is further confirmed as the bridging oxo dimer **6**. Therefore, benzene 10 ml was added to the grey mixture, and then the mixture was heated to 80 °C to dissolve the poorly soluble product **6**, and filtered to remove insoluble graphite and unreacted RbC_8. Colourless crystals started to form upon cooling the clear pale yellow filtrate, which was left to further crystallise at 10 °C for 16 h. After decanting the mother liquor, crystalline solid of **6** was dried *in vacuo*. Yield: 0.08 g, 8% (based on thorium). Anal. Calcd for $C_{66}H_{150}N_8O_2Rb_2Si_6Th_2$: C, 41.91; H, 7.99; N, 5.92%. Found: C, 42.42; H, 8.35; N, 5.95%. ATR-IR *v*/cm^{-1}: 2938 (br, m), 2860 (s), 1459(m), 1236 (s), 1145 (s), 1128 (s), 1057 (s), 984 (s), 927 (vs), 878 (s), 812 (w), 737 (vs), 669 (s), 625 (m), 596 (s), 566 (w), 538 (w), 511 (w), 436 (w). Similar the previously reported thorium bridging parent imido dimer [{Th(Tren^{TIPS})(μ-NHRb)}$_2$][11], complex **6** is poorly soluble in aromatic solvents (benzene or toluene), and rapidly decomposes in the polar solvents (THF or pyridine), and is therefore not amenable to NMR spectroscopic analysis.

Thermolysis of 5

In a glove box, complex **5** (0.02 g) was weighed into a small vial, and then 0.5 ml C_6D_6 was added, forming a yellow slurry this is due to **5** is only partially soluble in benzene once isolated as a pure yellow solid. The suspension was filtered into a J. Young NMR tube to give a yellow solution. The NMR tube was taken out of the glove box and heated at 80 °C in an oil bath for 8 hours. Monitoring the reaction by ^{1}H NMR showed that **5** slowly decomposed to **4** at 80 °C (See Figure S17). Upon cooling to room temperature, colourless crystals was formed and confirmed as the bridging dithorium oxo species **6** by XRD cell check. The presumed by-product for mass-balance is phosphorus-dicyclopentadiene species [$H_4C_4P_6$] via Diels-Alder

cycloaddition from these protonated phosphorus-heterocycles [$H_2C_2P_3$], although we were not able to isolate any organic species from this reaction.

Preparation of [{Th(Tren^{TIPS})(μ-OCs)}$_2$] (7)

Benzene (40 ml) was added slowly to a pre-cooled (–78 °C) mixture of **2** (0.90 g, 1.00 mmol) and CsC_8 (0.46 g, 2.00 mmol). The frozen mixture was left to thaw to room temperature and stirred for 2 days, during which time the formation of black graphite was observed, and the mixture turned into a yellow slurry. The mixture was then filtered to give a yellow solution. Removal of the volatiles afforded a dark yellow residue. ^{1}H NMR of the crude product also indicates the formation of **4**. Pentane 5 ml was added to the residue, forming a yellow slurry with off-white solid **7**. The off-white was collected by filtration and washed with pentane (5 x 5 mL), and dried *in vacuo*. Yield: 0.28 g, 28% (based on thorium). Colourless crystals suitable for an X-ray diffraction study were obtained by cooling a hot solution in benzene (80 °C) at room temperature as **7** is poorly soluble in benzene at room temperature. Anal. Calcd for $C_{66}H_{150}N_8O_2Cs_2Si_6Th_2$: C, 39.91; H, 7.61; N, 5.64%. Found: C, 39.52; H, 7.39; N, 5.87%. ^{1}H NMR (400 MHz, C_6D_6, 353 K): δ (ppm) 1.44 (d, 108H, CH(C*H*$_3$)$_2$), 1.52-1.60 (m, 18H, C*H*(CH$_3$)$_2$), 2.64 (t, $^3J_{HH}$ = 4.6 Hz, 12H, C*H*$_2$CH$_2$), 3.64 (t, $^3J_{HH}$ = 4.6 Hz, 12H, CH$_2$C*H*$_2$). ATR-IR *v*/cm^{-1}: 2935 (br, m), 2860 (s), 1458(m), 1275 (m), 1239 (m), 1137 (m), 1063 (s), 1011 (w), 985 (w), 931 (vs), 880 (s), 737 (vs), 667 (s), 628 (m), 579 (m), 538 (w), 508 (w), 466 (w). Similar the previously reported thorium bridging parent imido dimer [{Th(Tren^{TIPS})(μ-NHCs)}$_2$][11], complex **7** is poorly soluble in aromatic solvents (benzene or toluene), and rapidly decomposes in the polar solvents (THF or pyridine), and is therefore not amenable to ^{13}C and ^{29}Si NMR spectroscopic analysis, although a quite clean ^{1}H NMR spectrum (Figure S18) could be obtained at high temperature (80 °C).

Molecular Structure

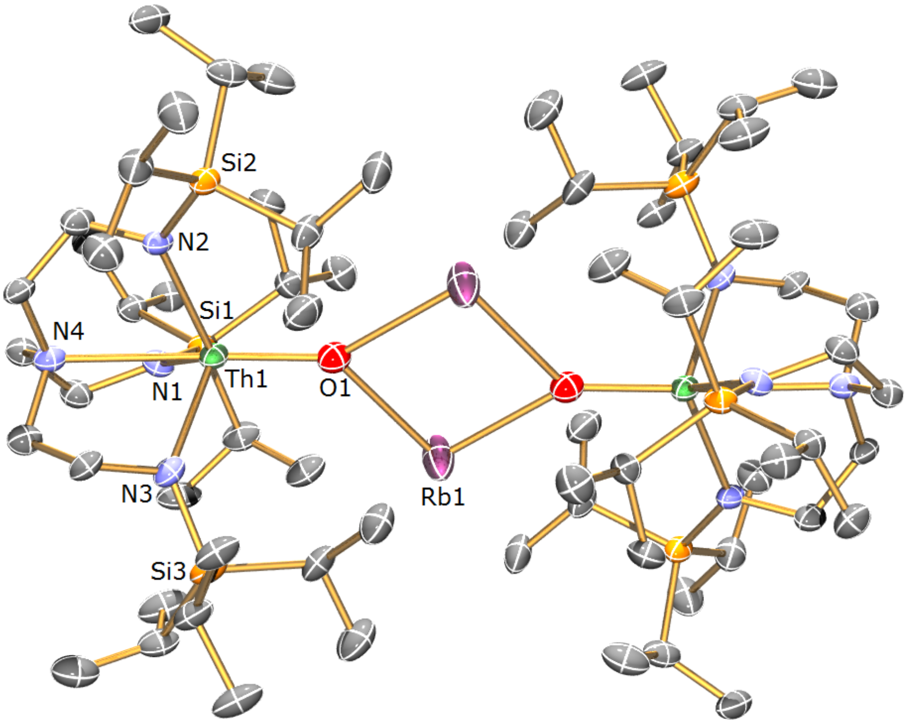

Figure S1. Molecular structure of **6** at 150 K. Displacement ellipsoids are set at 40% and hydrogen atoms are omitted for clarity.

ATR-IR Spectra

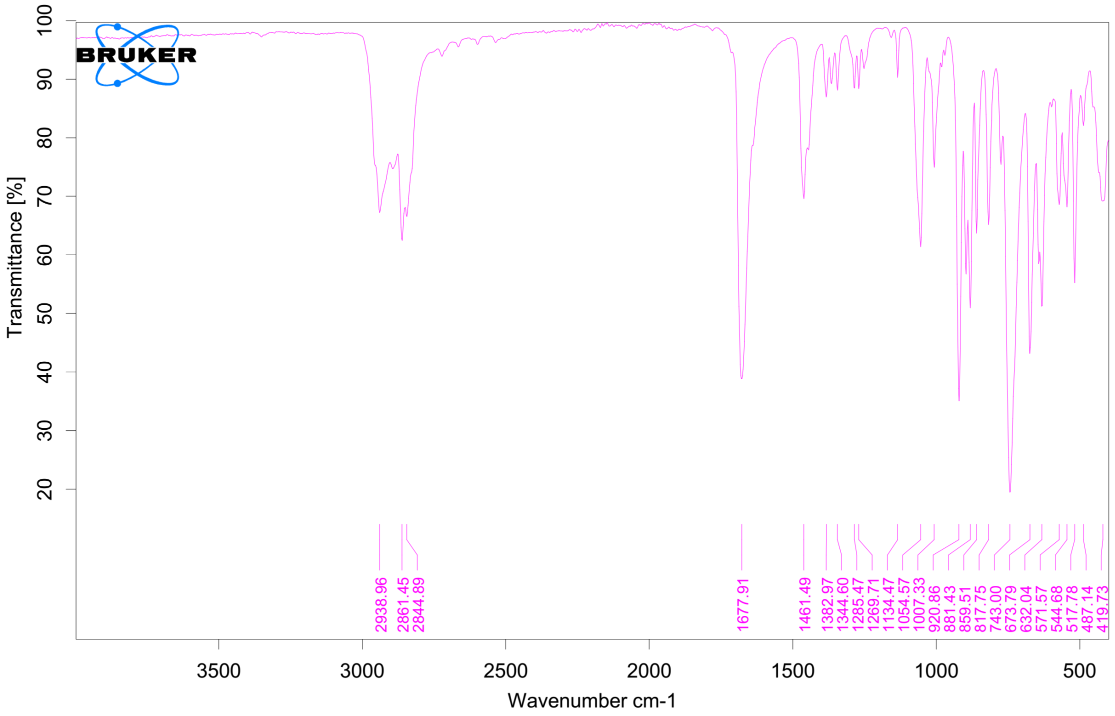

Figure S2. ATR-IR spectrum of **2**.

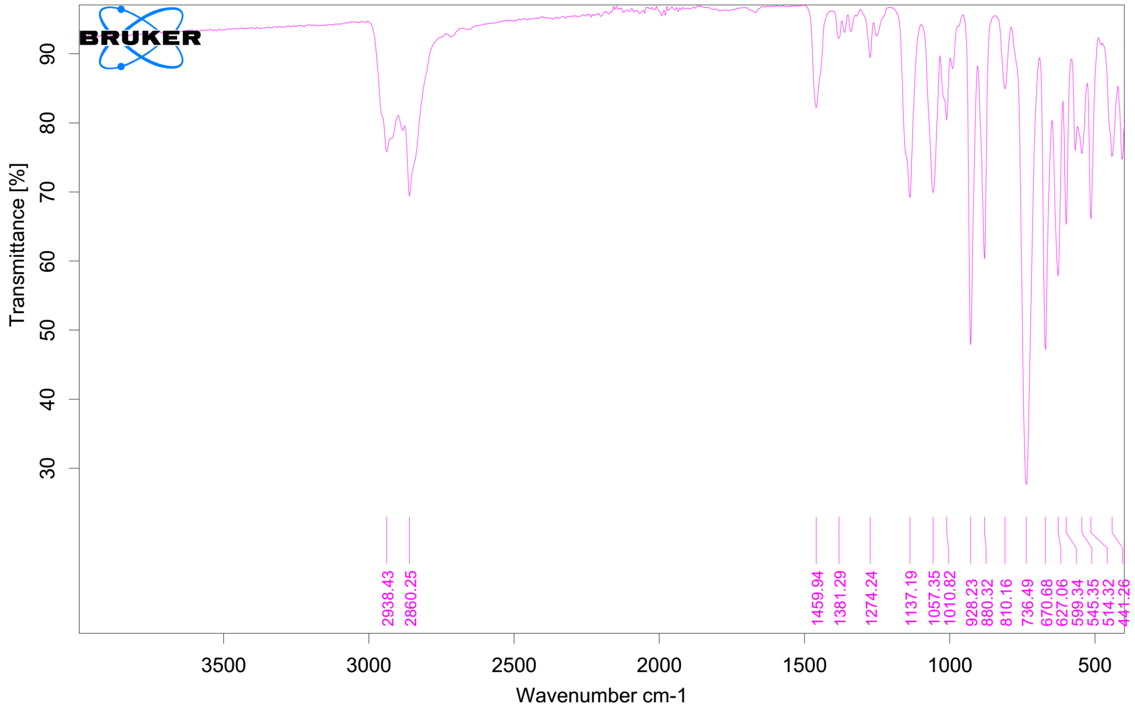

Figure S3. ATR-IR spectrum of **5**.

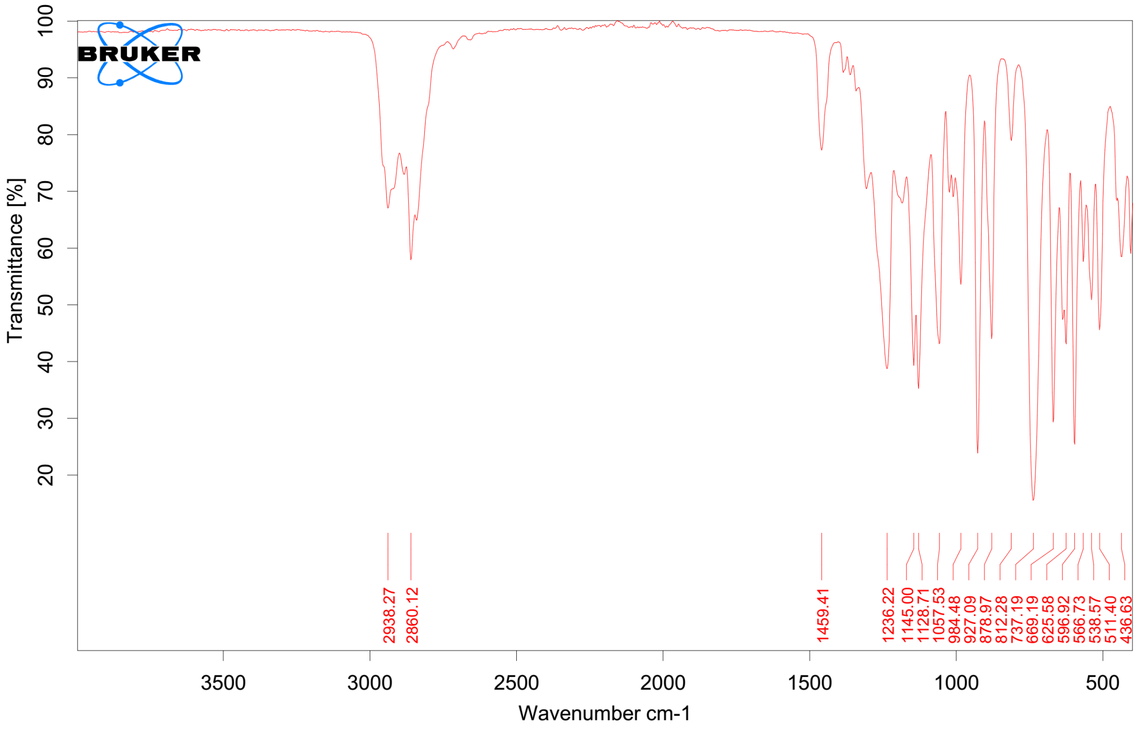

Figure S4. ATR-IR spectrum of **6**.

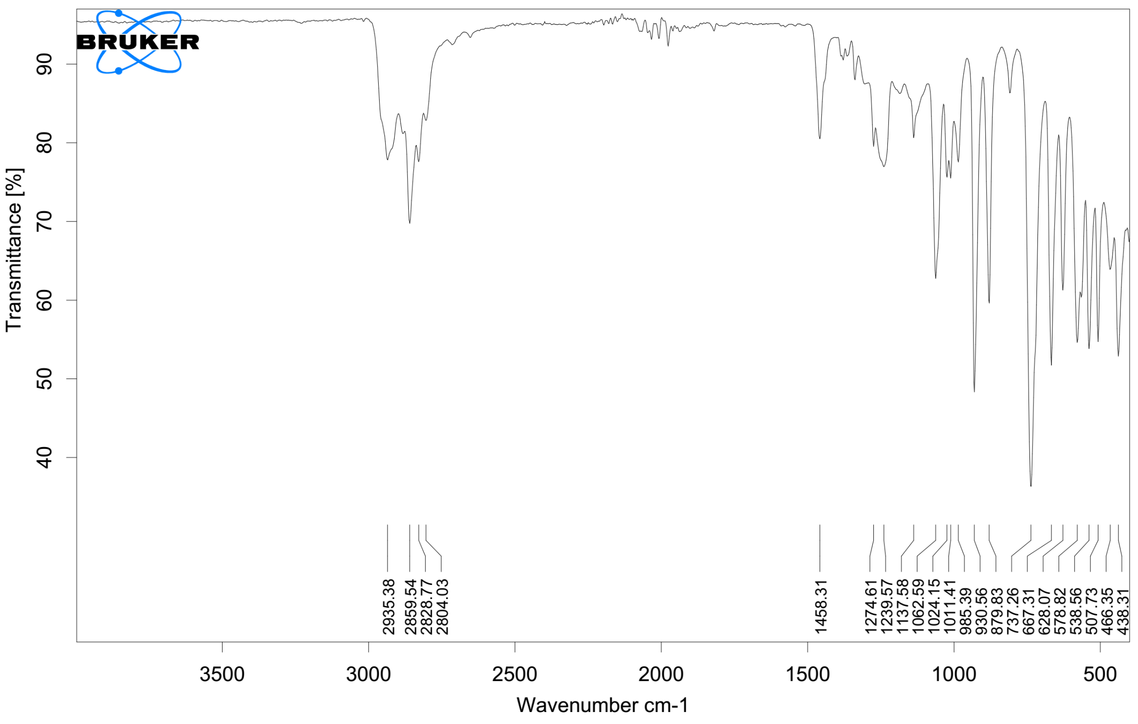

Figure S5. ATR-IR spectrum of **7**.

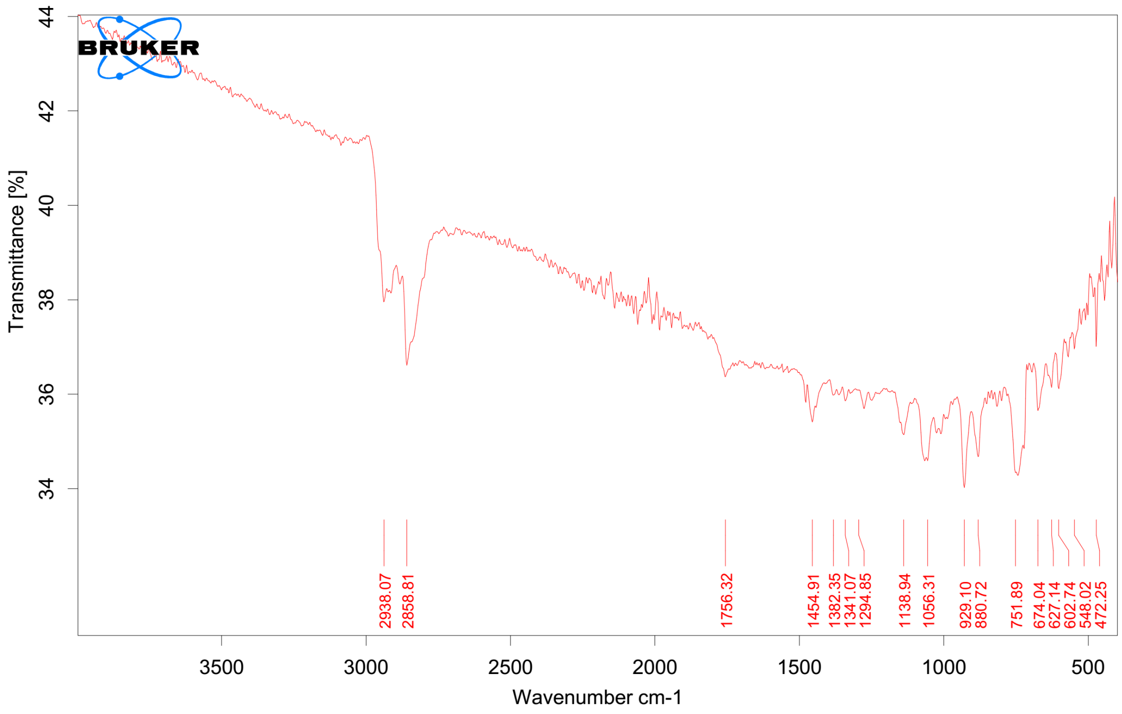

Figure S6. ATR-IR spectrum of insoluble graphite mixture from reduction of **2** with RbC_8.

NMR Spectra

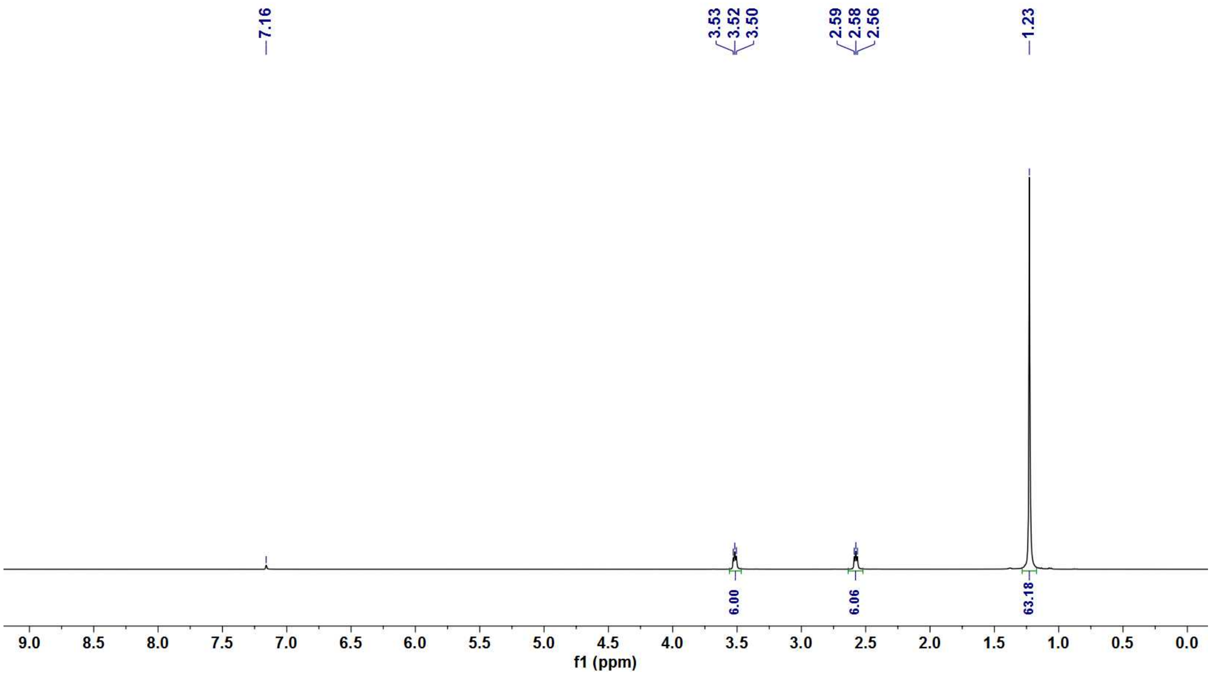

Figure S7. ^{1}H NMR (400 MHz, C_6D_6, 298 K) of **2**.

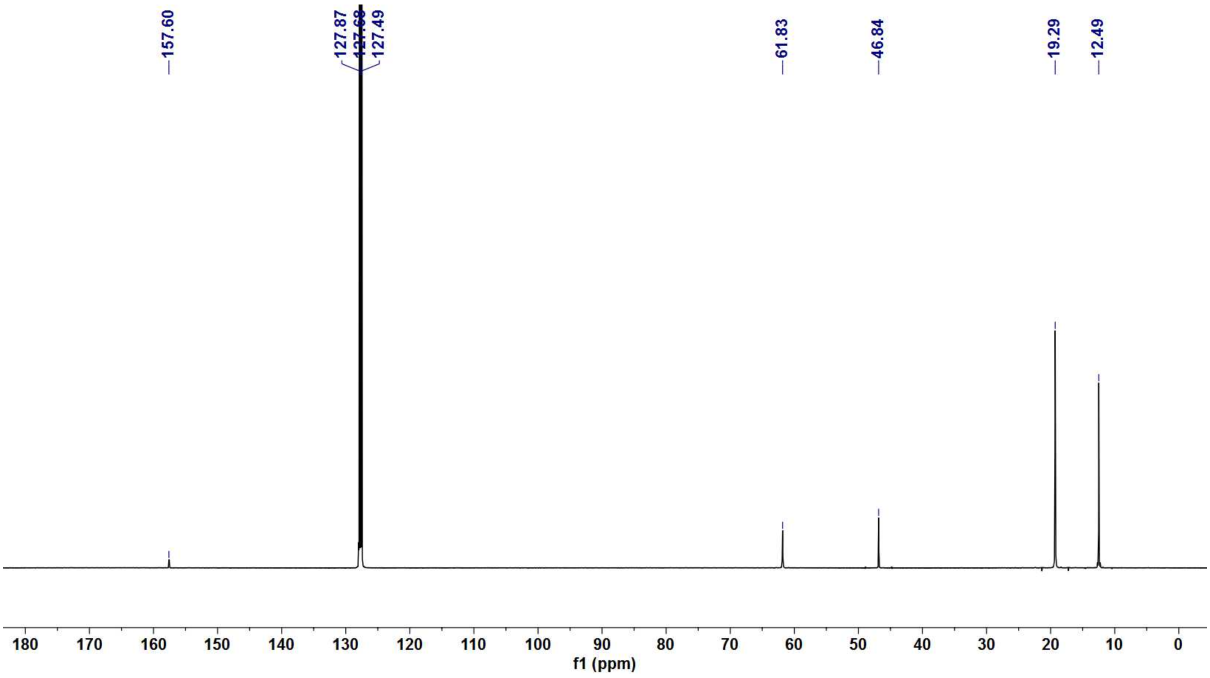

Figure S8. $^{13}C\{^1H\}$ NMR (101 MHz, C_6D_6, 298 K) of **2**.

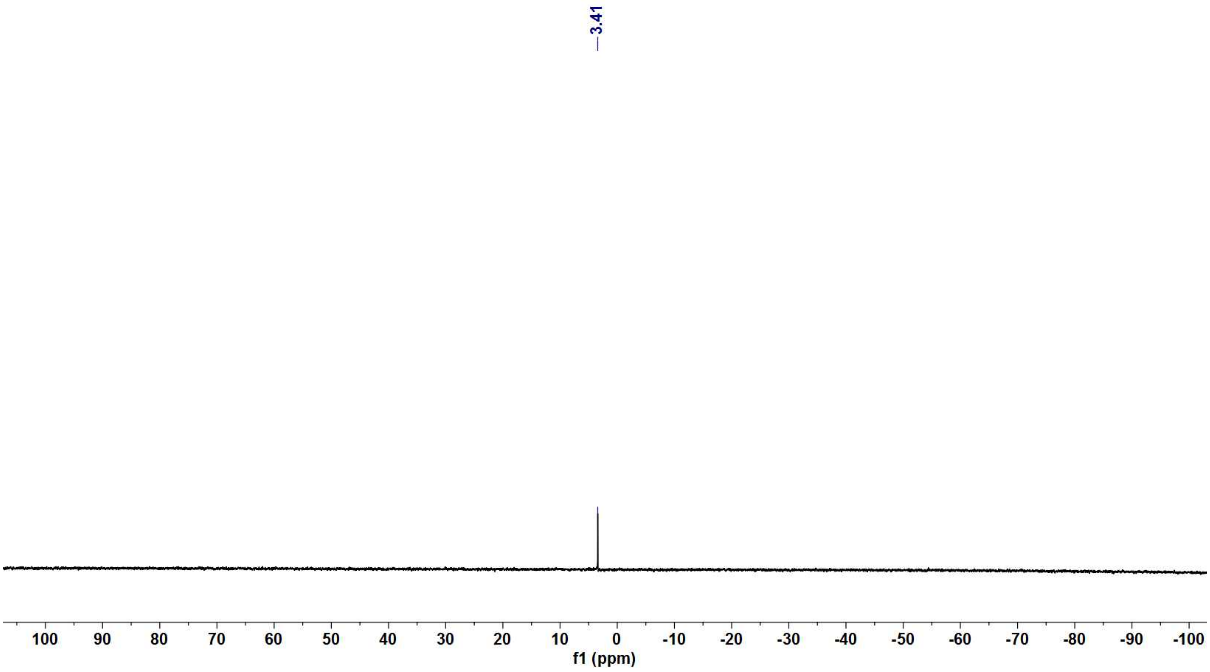

Figure S9. $^{29}Si\{^1H\}$ NMR (79 MHz, C_6D_6, 298 K) of **2**.

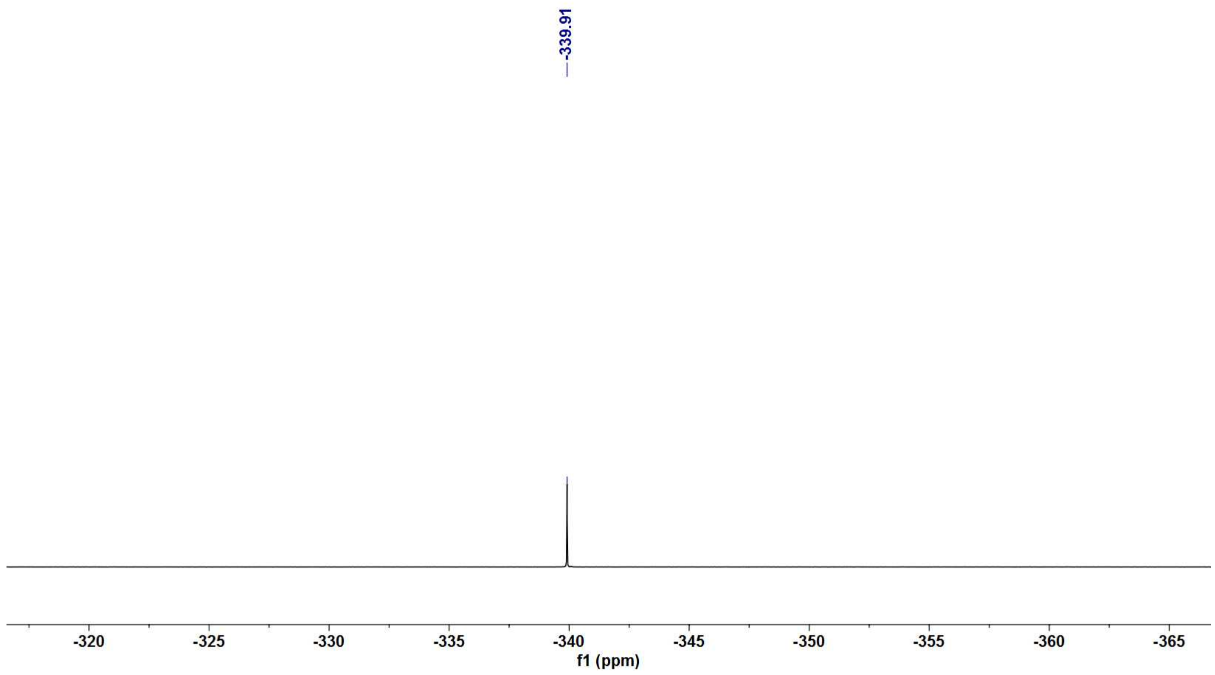

Figure S10. $^{31}P\{^{1}H\}$ NMR (162 MHz, C_6D_6, 298 K) of **2**.

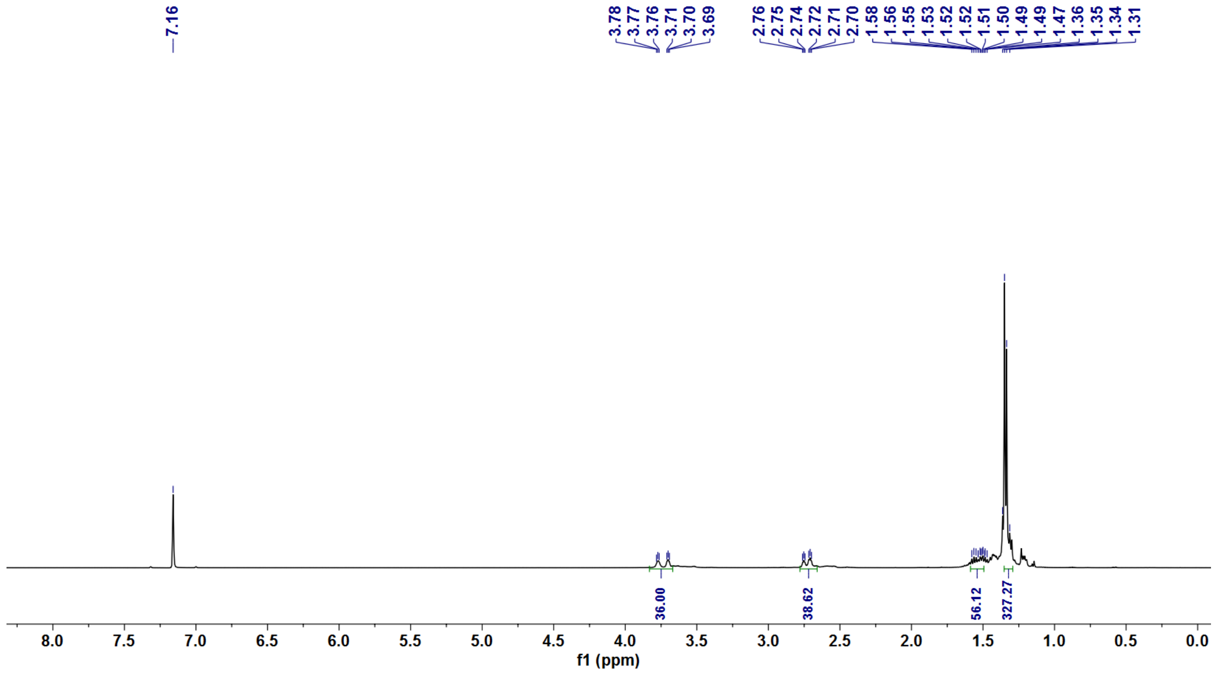

Figure S11. ^{1}H NMR (400 MHz, C_6D_6, 298 K) of **5**.

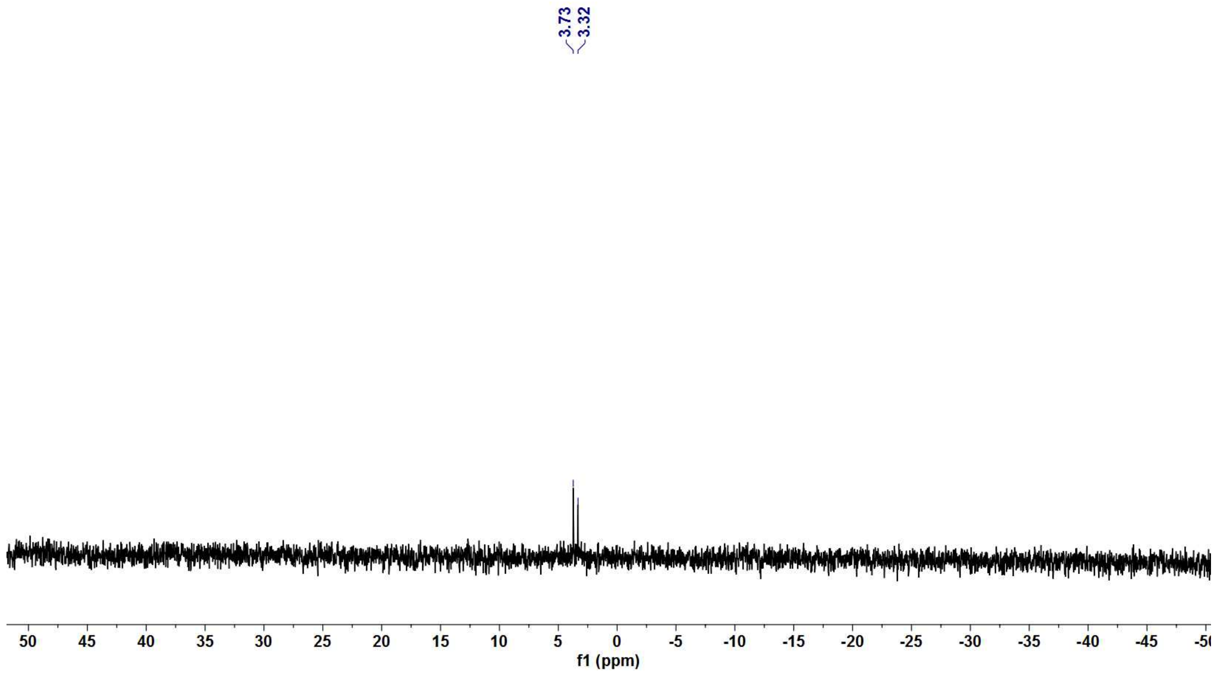

Figure S12. $^{29}Si\{^{1}H\}$ NMR (79 MHz, C_6D_6, 298 K) of **5**.

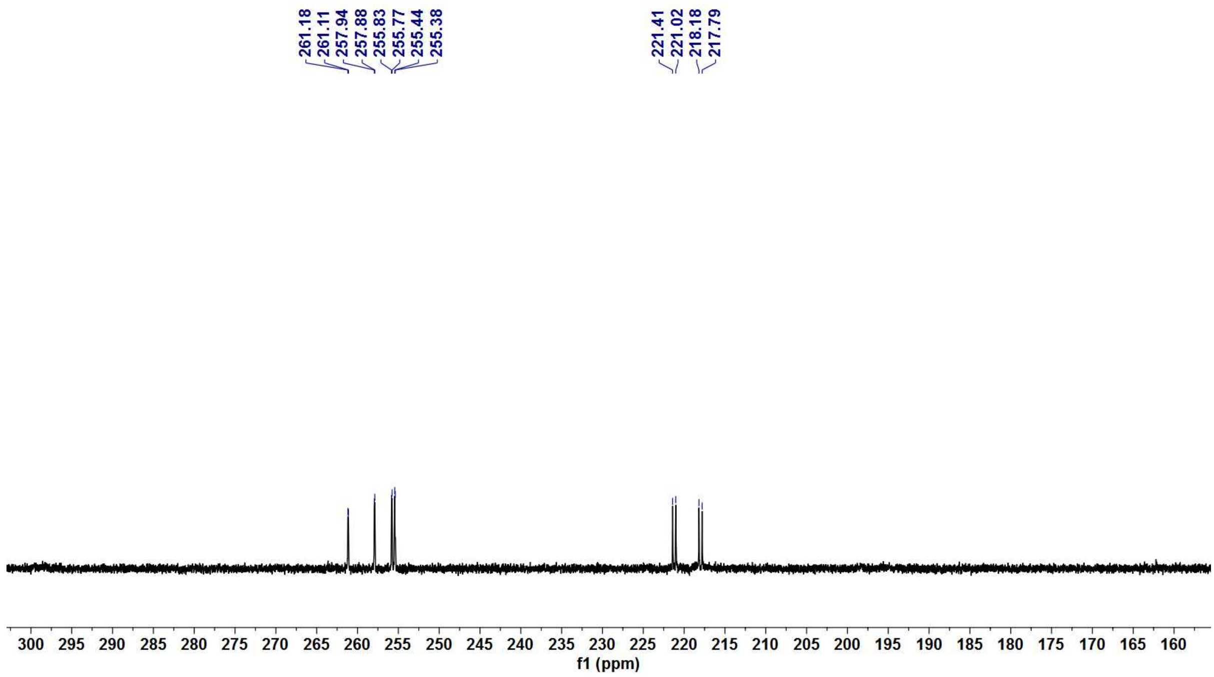

Figure S13. $^{31}P\{^{1}H$ decoupled$\}$ NMR (162 MHz, C_6D_6, 298 K) of **5**.

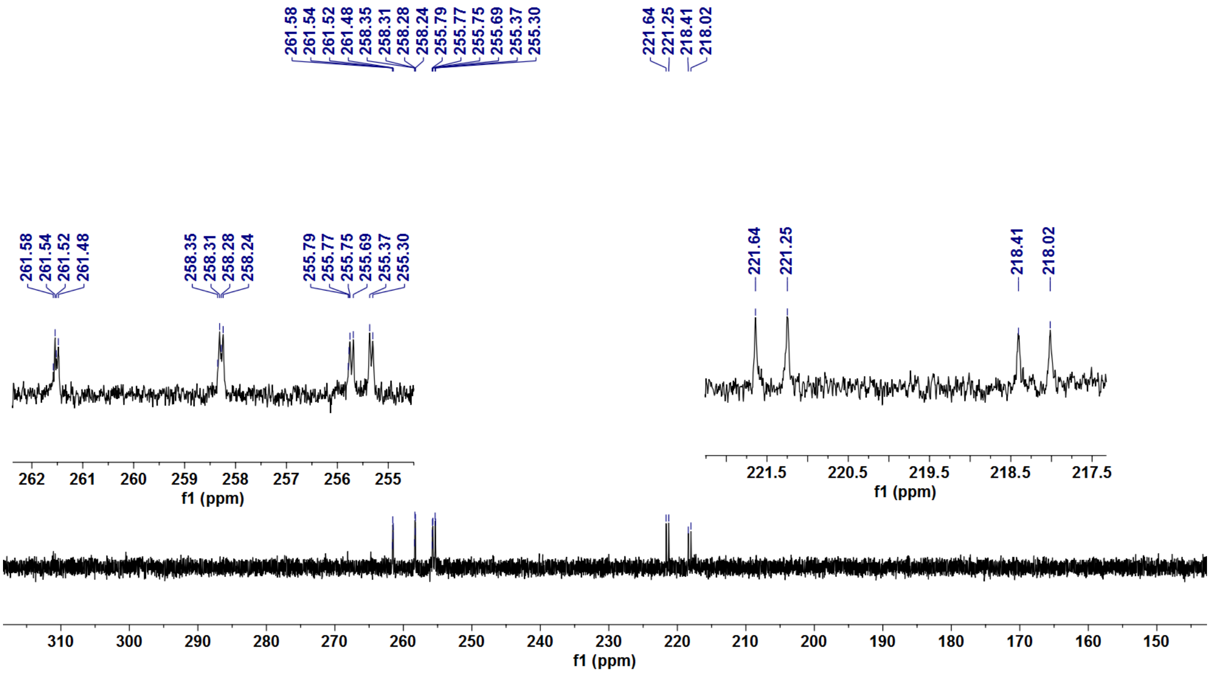

Figure S14. ^{31}P{Non-decoupled} NMR (162 MHz, C_6D_6, 298 K) of **5**.

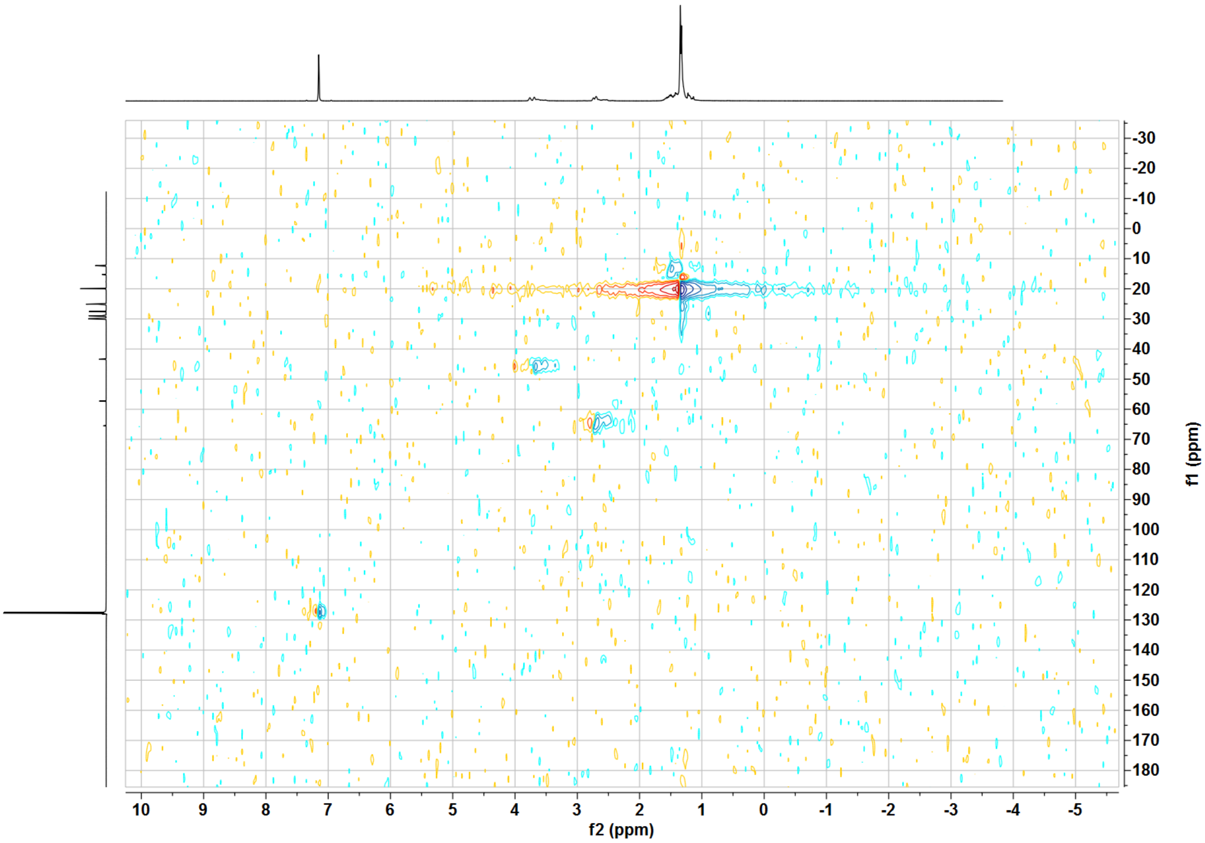

Figure S15. ^{1}H-^{13}C HSQC NMR (C_6D_6, 298 K) of **5**.

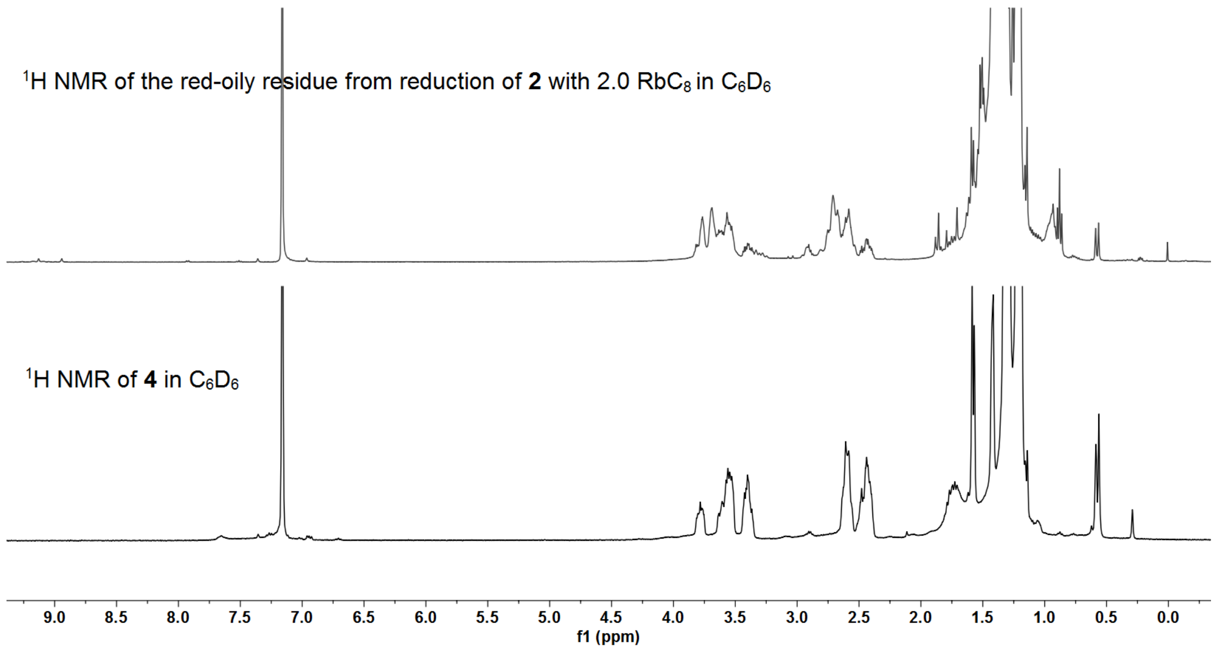

Figure S16. ^{1}H NMR of crude product from reduction of **2** with RbC_8 and **4**.

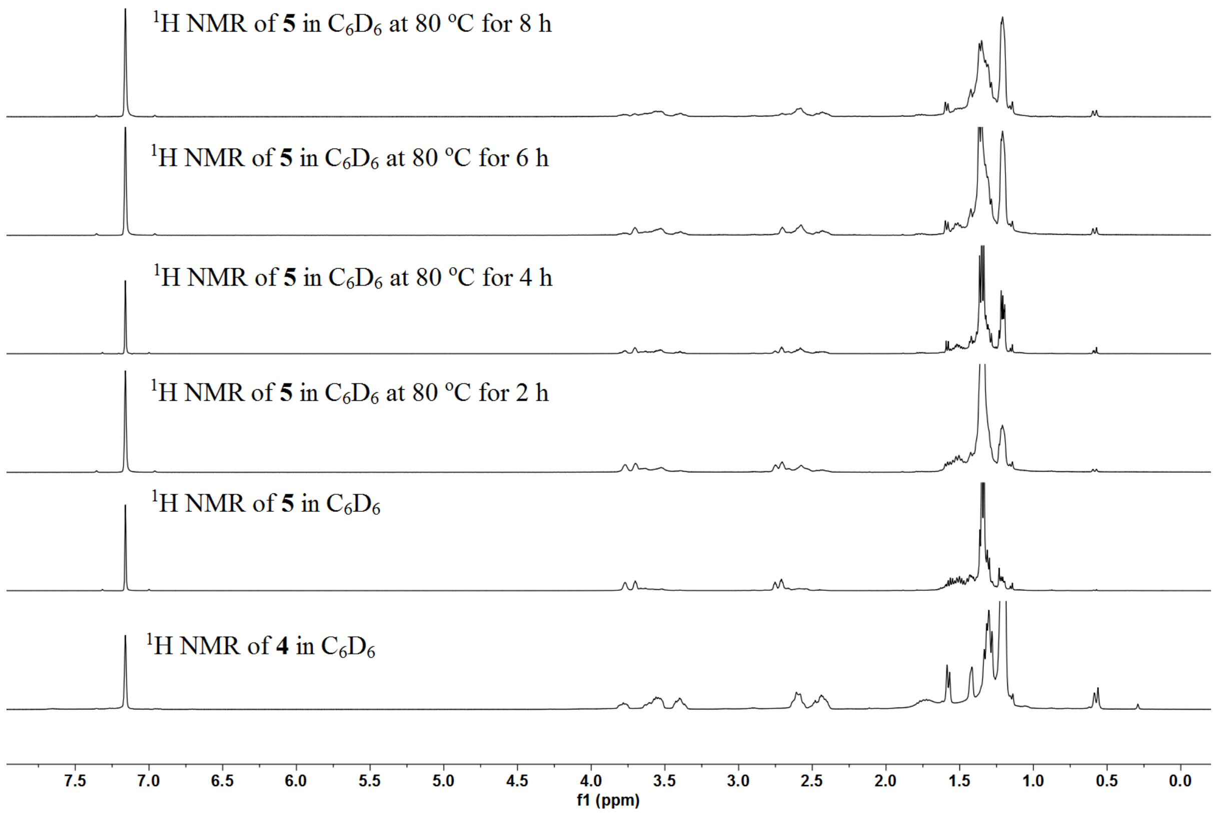

Figure S17. Thermolysis of **5** in C_6D_6 at 80 °C monitored by ^{1}H NMR.

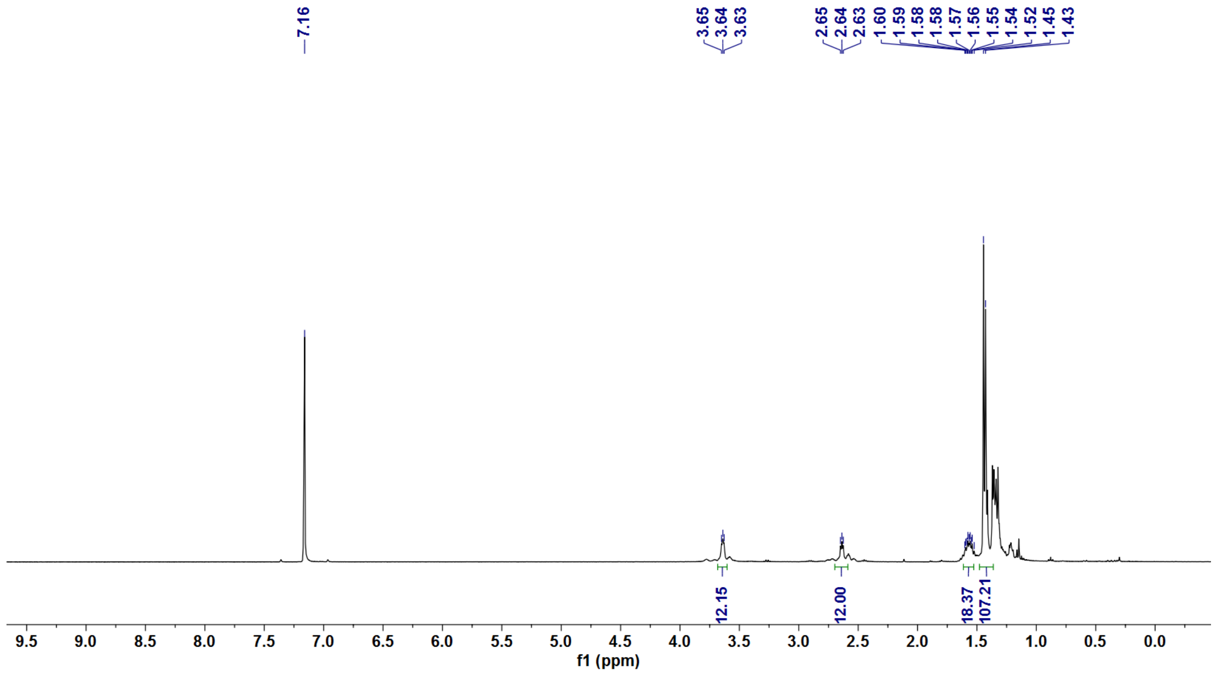

Figure S18. ^{1}H NMR of **7** in C_6D_6 at 80 °C.

General Computational Details

Geometry optimisations on **2** and **2**$^-$ were performed using coordinates derived from the crystal structure of **2**. No constraints were imposed on the structures during the geometry optimisations other than closed-shell species were restricted and open-shell were unrestricted calculations. A restricted single point energy calculation was performed on **6** using coordinates derived from its crystal structure. The calculations were performed using the Amsterdam Density Functional (ADF) suite version 2012 with standard convergence criteria.[12,13] The DFT geometry optimisations employed Slater type orbital (STO) triple-ζ-plus polarisation all electron basis sets (from the Dirac and ZORA/TZP database of the ADF suite). Scalar relativistic approaches (spin-orbit neglected) were used within the ZORA Hamiltonian[14-16] for the inclusion of relativistic effects and the local density approximation (LDA) with the correlation potential due to Vosko et al was used in all of the calculations.[17]

Generalised gradient approximation corrections were performed using the functionals of Becke and Perdew.[18,19] MOLEKEL[20] was used to prepare the three-dimensional plots of the electron density. Frequencies were computed using the analytical frequencies routine in ADF.

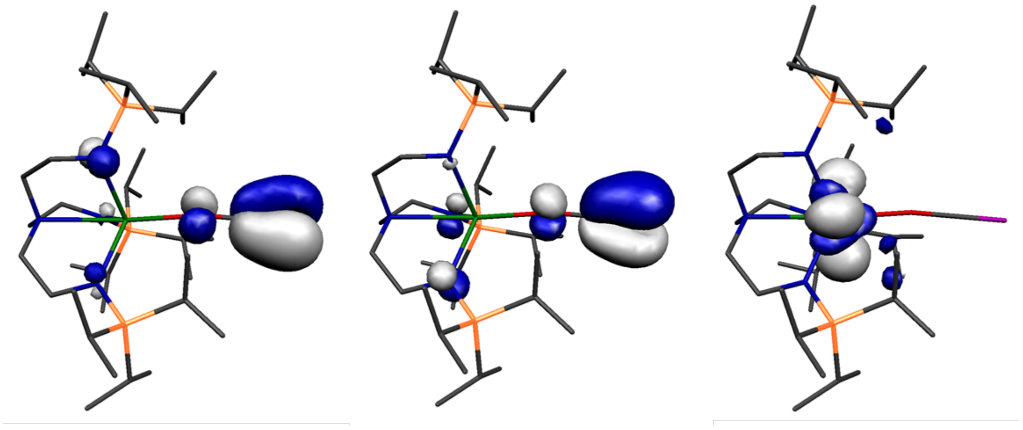

Figure S19. Kohn Sham molecular orbital representations of the principal frontier orbitals of **2** with hydrogen atoms omitted for clarity. Left to right: HOMO−1 (230a, −4.869 eV), HOMO (231a, −4.865 eV), LUMO (232a, −1.513 eV).

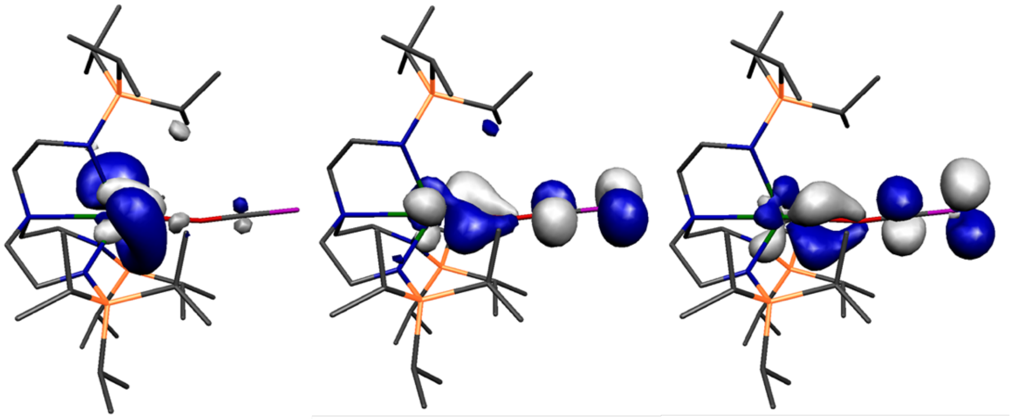

Figure S20. Kohn Sham molecular orbital representations of the principal frontier orbitals of **2**$^{-}$ with hydrogen atoms omitted for clarity. Left to right: HOMO (232a, 1.287 eV), LUMO (233a, 1.741 eV), LUMO−1 (234a, 1.789 eV).

Table S1. Coordinates and final energy for a single point energy calculation on geometry optimised 2

1.C	2.966567	1.421833	-5.441270
2.C	1.181756	-0.250076	-4.779177
3.C	1.736328	1.168895	-4.544652
4.C	1.880762	4.465037	-3.446610
5.C	2.674207	3.433439	-2.618515
6.C	-1.852329	1.343796	-2.638709
7.C	1.036794	-4.145866	-2.496788
8.C	-0.634527	2.225620	-2.381981
9.C	4.766452	0.766422	-2.182488
10.C	-1.926990	-1.730455	-1.944822
11.C	-0.260141	-4.615884	-1.812007
12.C	-0.229024	-6.140596	-1.572508
13.C	2.996196	-0.995385	-1.835213
14.C	3.298367	0.515028	-1.777207
15.C	-2.950136	-0.632734	-1.676329
16.C	2.852008	3.929728	-1.171075
17.C	-3.407790	-4.744841	-0.534303
18.C	-3.117849	1.544597	-0.551832
19.C	-2.330251	-4.320459	0.488929
20.C	-3.019688	1.162482	0.920561
21.C	0.697121	-3.441207	1.005921
22.C	1.363567	-4.791224	1.343336
23.C	-2.989350	-3.468659	1.591442
24.C	-1.488026	4.563123	1.731220
25.C	3.018987	-0.556082	1.853773
26.C	1.426572	2.555781	2.327376
27.C	0.330577	-2.716605	2.318657
28.C	-1.967872	3.719335	2.927750
29.C	0.549264	1.700565	3.264865
30.C	-1.709930	4.459478	4.257441
31.C	-1.985590	-0.520615	4.388618
32.C	-2.278237	0.990979	4.373306
33.C	-3.798948	1.244808	4.427380
34.C	0.994332	1.897949	4.729495
35.H	2.719346	1.254525	-6.503703
36.H	0.831158	-0.374036	-5.817585
37.H	3.348152	2.449527	-5.351073
38.H	3.794083	0.739494	-5.191903
39.H	0.952666	1.877958	-4.877164
40.H	1.734131	4.147652	-4.489840
41.H	1.952429	-1.015724	-4.606416
42.H	0.339880	-0.481560	-4.110735
43.H	2.408338	5.433536	-3.471067
44.H	1.225914	-4.717876	-3.421051
45.H	-1.574878	0.575123	-3.371533
46.H	4.954631	0.484671	-3.229783

47.H	-0.367877	2.697435	-3.342624
48.H	3.681716	3.365121	-3.072323
49.H	0.887274	4.655327	-3.012351
50.H	-2.697626	1.923435	-3.059075
51.H	0.993644	-3.082564	-2.767571
52.H	-1.396613	-1.517227	-2.891778
53.H	-0.100037	-6.679761	-2.526337
54.H	3.247031	-1.414335	-2.820437
55.H	-1.084620	-4.426450	-2.526673
56.H	-3.655517	-0.515171	-2.522582
57.H	5.058494	1.819039	-2.063609
58.H	-2.490915	-2.658905	-2.136114
59.H	1.913640	-4.285498	-1.845681
60.H	5.444049	0.166383	-1.553603
61.H	-0.915803	3.063614	-1.717950
62.H	1.932677	-1.247153	-1.670892
63.H	-3.005107	-5.337045	-1.367504
64.H	3.299642	4.937724	-1.147257
65.H	-1.151946	-6.515602	-1.107300
66.H	0.606990	-6.434629	-0.921225
67.H	-3.918587	-3.869382	-0.965715
68.H	3.579039	-1.544093	-1.079565
69.H	-4.166006	1.525219	-0.909885
70.H	-3.532676	-0.911248	-0.787558
71.H	1.882487	3.989408	-0.653549
72.H	3.501039	3.269158	-0.578674
73.H	3.215726	0.820247	-0.717126
74.H	-2.742882	2.570322	-0.668922
75.H	-4.186988	-5.352935	-0.044252
76.H	1.813425	-5.262641	0.459320
77.H	1.473445	-2.832155	0.502729
78.H	-1.957057	-5.248616	0.966100
79.H	-1.664005	4.054501	0.772456
80.H	-3.515568	0.187761	1.085800
81.H	-3.288399	-2.481238	1.205275
82.H	1.113420	2.520404	1.267310
83.H	-3.625871	1.895726	1.480920
84.H	0.644260	-5.505111	1.775335
85.H	-2.011079	5.533829	1.698307
86.H	-0.411094	4.778160	1.791707
87.H	-3.899469	-3.959917	1.975584
88.H	2.168639	-4.646048	2.082152
89.H	-0.286495	-1.809050	2.180377
90.H	2.478487	2.234053	2.371339
91.H	-2.325442	-3.295847	2.446855
92.H	1.387047	3.618038	2.610269
93.H	-3.066653	3.621531	2.828666
94.H	1.235917	-2.419156	2.869529
95.H	-0.261224	-3.369164	2.976858
96.H	0.763696	0.637295	3.034437

97.H	-2.361604	-1.004663	3.474713
98.H	-4.312711	0.843602	3.540183
99.H	-2.201782	5.447130	4.261116
100.H	-0.910062	-0.737040	4.457349
101.H	-0.634476	4.634048	4.417476
102.H	-4.043946	2.314812	4.498215
103.H	2.072182	1.692020	4.829662
104.H	0.817972	2.928253	5.073975
105.H	-2.086278	3.902368	5.128342
106.H	-2.474883	-1.013155	5.245849
107.H	-4.244869	0.749010	5.306270
108.H	-1.847744	1.420651	5.298230
109.H	0.466657	1.222201	5.417197
110.N	0.479443	1.436966	-1.802389
111.N	-2.268017	0.656215	-1.387358
112.N	-0.973982	-1.861361	-0.818607
113.N	-1.609780	1.129119	1.367849
114.O	2.026334	-0.350684	1.111624
115.P	4.271406	-0.815842	2.792170
116.Si	2.014774	1.620939	-2.683107
117.Si	-0.731807	-3.536502	-0.279575
118.Si	-1.335874	1.891856	2.948093
119.Th	-0.017795	0.087725	0.022650

Energy: –645.43257306 eV

Table S2. Coordinates and final energy for a single point energy calculation on geometry optimised 2^-

1.C	3.081675	1.404294	-5.573019
2.C	1.252866	-0.253214	-4.973164
3.C	1.838486	1.145201	-4.700432
4.C	1.852865	4.401635	-3.480606
5.C	2.670962	3.359402	-2.691323
6.C	-1.896687	1.301680	-2.657532
7.C	0.993390	-4.116030	-2.513954
8.C	-0.601429	2.098209	-2.513748
9.C	4.832602	0.792126	-2.284360
10.C	-1.961232	-1.728303	-1.898054
11.C	-0.297212	-4.595820	-1.825648
12.C	-0.270446	-6.127315	-1.634664
13.C	3.146565	-1.059948	-1.968865
14.C	3.373899	0.461808	-1.904357
15.C	-2.982559	-0.629160	-1.623656
16.C	2.839484	3.815830	-1.229371
17.C	-3.423709	-4.785798	-0.569855
18.C	-3.105530	1.571764	-0.534991
19.C	-2.359159	-4.357223	0.465041

20.C	-3.070299	1.165328	0.941827
21.C	0.673264	-3.557191	1.033090
22.C	1.332146	-4.935246	1.246206
23.C	-3.042527	-3.532083	1.572335
24.C	-1.532404	4.529718	1.787702
25.C	3.053872	-0.468696	1.920473
26.C	1.387535	2.594920	2.456274
27.C	0.330630	-2.947791	2.407567
28.C	-2.010263	3.690147	2.988072
29.C	0.505237	1.681675	3.330688
30.C	-1.737480	4.436061	4.310050
31.C	-1.972148	-0.520812	4.509997
32.C	-2.304736	0.981767	4.463114
33.C	-3.832185	1.190286	4.483530
34.C	0.929541	1.811089	4.808765
35.H	2.849069	1.280662	-6.646724
36.H	0.916930	-0.354706	-6.020771
37.H	3.481003	2.421277	-5.438098
38.H	3.893301	0.697889	-5.336733
39.H	1.069513	1.877927	-5.017942
40.H	1.717887	4.122140	-4.536926
41.H	1.998283	-1.042499	-4.791971
42.H	0.396876	-0.462064	-4.315772
43.H	2.352828	5.387207	-3.462416
44.H	1.174736	-4.664318	-3.456468
45.H	-1.725884	0.502453	-3.390899
46.H	5.048764	0.545026	-3.335844
47.H	-0.397701	2.540116	-3.508260
48.H	3.678505	3.330674	-3.149907
49.H	0.852815	4.543134	-3.043540
50.H	-2.722768	1.938829	-3.037553
51.H	0.948920	-3.044806	-2.750057
52.H	-1.443043	-1.507978	-2.852972
53.H	-0.163789	-6.641150	-2.607699
54.H	3.333981	-1.455776	-2.979095
55.H	-1.127168	-4.383689	-2.528046
56.H	-3.708642	-0.523602	-2.458144
57.H	5.075196	1.854553	-2.136221
58.H	-2.533101	-2.653805	-2.092949
59.H	1.875686	-4.272187	-1.873420
60.H	5.529071	0.207900	-1.659714
61.H	-0.783808	2.959239	-1.837616
62.H	2.117307	-1.347795	-1.698258
63.H	-3.007425	-5.366050	-1.405513
64.H	3.244774	4.842918	-1.173128
65.H	-1.184420	-6.513051	-1.159241
66.H	0.577354	-6.445501	-1.010290
67.H	-3.937517	-3.911679	-1.000946
68.H	3.820658	-1.583495	-1.271847
69.H	-4.151521	1.613959	-0.911482

70.H	-3.543452	-0.897796	-0.718060
71.H	1.871980	3.808394	-0.701779
72.H	3.519523	3.162634	-0.663756
73.H	3.259936	0.750277	-0.841572
74.H	-2.670129	2.576320	-0.626784
75.H	-4.204360	-5.406513	-0.093232
76.H	1.784184	-5.331983	0.326394
77.H	1.441248	-2.900125	0.581136
78.H	-1.973983	-5.286393	0.932355
79.H	-1.660124	3.995699	0.834455
80.H	-3.617616	0.209725	1.066210
81.H	-3.365032	-2.551002	1.189616
82.H	1.084153	2.597144	1.388971
83.H	-3.683299	1.916194	1.476595
84.H	0.610805	-5.683348	1.615920
85.H	-2.082226	5.487113	1.725485
86.H	-0.462440	4.772567	1.868041
87.H	-3.939431	-4.050757	1.958668
88.H	2.134931	-4.856935	1.999022
89.H	-0.214463	-1.992390	2.320091
90.H	2.442880	2.281757	2.507536
91.H	-2.377386	-3.339897	2.423091
92.H	1.325603	3.642664	2.790217
93.H	-3.110712	3.597599	2.894818
94.H	1.251325	-2.750426	2.981142
95.H	-0.297767	-3.629136	3.003485
96.H	0.742316	0.634816	3.050578
97.H	-2.315054	-1.024353	3.593817
98.H	-4.309365	0.753378	3.592554
99.H	-2.210670	5.434970	4.309735
100.H	-0.891458	-0.704824	4.590798
101.H	-0.657345	4.592077	4.461335
102.H	-4.110276	2.254757	4.520014
103.H	2.009028	1.609856	4.906878
104.H	0.740752	2.824065	5.198880
105.H	-2.117305	3.890501	5.187460
106.H	-2.458370	-1.015928	5.370002
107.H	-4.286560	0.701458	5.364311
108.H	-1.898050	1.445768	5.382700
109.H	0.401885	1.099146	5.460114
110.N	0.503508	1.254846	-2.016599
111.N	-2.293543	0.660660	-1.376620
112.N	-1.001867	-1.852876	-0.781954
113.N	-1.696718	1.065249	1.457823
114.O	2.084758	-0.300067	1.151586
115.P	4.291120	-0.726278	2.900498
116.Si	2.043548	1.525266	-2.806924
117.Si	-0.753932	-3.517332	-0.266373
118.Si	-1.384577	1.849695	2.994243
119.Th	-0.004477	0.176451	0.043086

Energy: −645.70910207 eV

Table S3. Coordinates and final energy for a single point energy calculation on 6

C	-2.60069	-3.51997	-5.85299
C	5.30420	1.89614	-5.55524
C	-3.19696	-1.01627	-5.68569
C	-3.50533	-2.48929	-5.11135
C	-5.61448	3.21763	-4.25876
C	6.70244	0.32946	-4.11233
C	2.24269	0.46049	-4.44630
C	5.81033	1.56486	-4.11685
C	-4.71601	5.49331	-3.64489
C	-5.44310	-4.68460	-3.60650
C	-5.64876	-1.09119	-3.16659
C	-5.34375	4.20306	-3.03402
C	-2.43256	2.25109	-3.32383
C	3.58594	-1.21991	-3.24288
C	3.04190	0.23734	-3.18707
C	-4.17797	-4.33680	-2.86754
C	-0.82759	-1.41306	-3.14637
C	3.54361	3.19439	-2.93100
C	4.49688	4.34615	-2.54511
C	-0.92696	-3.89725	-2.61033
C	-1.70668	-2.60070	-2.54874
C	-6.79441	-1.30409	-2.14308
C	-1.58991	4.30665	-2.08964
C	-2.56912	3.09542	-2.09919
C	2.30898	3.26880	-2.09179
C	-7.20079	1.01470	-1.34966
C	-4.27742	-4.49977	-1.29631
C	6.32966	1.83451	-0.91851
C	3.62675	-4.43614	-0.96106
C	-6.42928	2.08239	-0.65806
C	7.24752	0.85533	-0.23671
C	-4.27804	4.92459	-0.30274
C	-5.64397	5.55203	-0.00394
C	5.64383	-5.55172	0.00397
C	-7.24754	-0.85535	0.23671
C	4.27803	-4.92461	0.30274
C	6.42917	-2.08224	0.65785
C	-6.32967	-1.83453	0.91851
C	-3.62676	4.43613	0.96106
C	7.20078	-1.01471	1.34966
C	4.27741	4.49976	1.29631
C	6.79454	1.30432	2.14310
C	1.58990	-4.30667	2.08964
C	-2.30899	-3.26882	2.09179

C	2.56937	-3.09552	2.09918
C	-4.49689	-4.34617	2.54511
C	1.70667	2.60069	2.54874
C	0.92690	3.89739	2.61056
C	4.17795	4.33678	2.86754
C	5.34374	-4.20308	3.03402
C	-3.54362	-3.19440	2.93100
C	5.64875	1.09117	3.16659
C	-3.04176	-0.23748	3.18695
C	5.44324	4.68446	3.60639
C	-3.58579	1.21977	3.24276
C	0.82758	1.41304	3.14637
C	4.71611	-5.49329	3.64501
C	2.43255	-2.25110	3.32383
C	-6.70246	-0.32947	4.11233
C	-5.81049	-1.56475	4.11696
C	5.61447	-3.21765	4.25877
C	-2.24279	-0.46034	4.44609
C	3.50532	2.48928	5.11135
C	-5.30421	-1.89615	5.55524
C	3.19695	1.01625	5.68569
C	2.60065	3.51975	5.85308
H	-2.78617	-3.48467	-6.81467
H	-3.25685	-1.02785	-6.66374
H	5.97328	2.44130	-6.01958
H	5.16040	1.06315	-6.05106
H	-1.65859	-3.30190	-5.69392
H	-2.78858	-4.42104	-5.51687
H	4.46094	2.39241	-5.49855
H	-4.44460	-2.70059	-5.38163
H	-2.29508	-0.74301	-5.41690
H	-3.85141	-0.38179	-5.32575
H	2.83405	0.39207	-5.22476
H	-6.38104	3.54234	-4.77591
H	-4.82175	3.18655	-4.83425
H	7.54084	0.53016	-4.57855
H	-5.28519	-4.62265	-4.57161
H	6.24343	-0.40658	-4.56809
H	-5.34319	5.89022	-4.28489
H	1.53852	-0.21809	-4.51033
H	-3.88136	5.26432	-4.10452
H	1.83650	1.35191	-4.42183
H	3.92231	-1.40868	-4.14365
H	-5.80674	2.31896	-3.91877
H	6.39719	2.32887	-3.84879
H	-2.68062	2.77712	-4.11267
H	-5.76224	-1.71797	-3.92440
H	-0.85491	-1.44709	-4.12551
H	3.26995	3.33576	-3.88238
H	-5.71438	-5.59809	-3.37764

H	-6.15300	-4.05925	-3.35084
H	-5.69129	-0.16831	-3.52259
H	-0.56028	-4.01535	-3.51153
H	6.89703	0.07033	-3.18756
H	-3.49309	-5.00006	-3.16924
H	-1.50398	1.94979	-3.41111
H	-4.52927	6.13605	-2.92914
H	5.37009	4.20231	-2.96594
H	-3.02311	1.47254	-3.25154
H	4.12190	5.19744	-2.85310
H	-6.23756	4.46147	-2.66737
H	2.86434	-1.84636	-3.02569
H	-7.66972	-1.16811	-2.58511
H	-1.85085	4.94310	-2.78770
H	0.09922	-1.50771	-2.84213
H	-1.18638	-0.55448	-2.83927
H	-7.15767	1.15214	-2.32911
H	4.31341	-1.32190	-2.59442
H	-1.52203	-4.64672	-2.39915
H	1.60936	2.70933	-2.48935
H	2.39643	0.27635	-2.42416
H	-0.67806	3.99076	-2.25980
H	-6.76408	-2.22964	-1.79243
H	1.99915	4.19752	-2.04811
H	6.75410	2.15879	-1.75206
H	-0.19398	-3.86898	-1.96057
H	3.56701	-5.17354	-1.60388
H	4.60348	4.36948	-1.57132
H	-8.15042	1.05577	-1.07294
H	-1.81941	-2.39514	-1.57665
H	4.16199	-3.71064	-1.34505
H	-4.75886	-5.32636	-1.08353
H	-2.24164	2.50883	-1.35849
H	-1.62492	4.74806	-1.21551
H	5.58108	-6.10146	-0.80511
H	-5.91805	6.11292	-0.75921
H	-6.81000	2.96391	-0.89897
H	7.44899	0.10879	-0.85484
H	2.50883	2.94837	-1.18746
H	-4.75909	-3.73460	-0.91829
H	-3.37581	-4.53920	-0.91439
H	-3.69573	5.64696	-0.67596
H	2.72649	-4.10589	-0.75814
H	6.52550	-1.96809	-0.32062
H	6.18125	2.61409	-0.32681
H	6.30617	-4.84272	-0.13354
H	8.10010	1.30581	-0.01260
H	-8.10011	-1.30583	0.01260
H	-6.30603	4.84258	0.13343
H	-6.52566	1.96819	0.32074

H	-6.18126	-2.61410	0.32681
H	5.91804	-6.11293	0.75921
H	-5.58111	6.10123	0.80521
H	3.69572	-5.64698	0.67596
H	-7.44916	-0.10868	0.85496
H	6.80999	-2.96392	0.89897
H	8.15041	-1.05578	1.07294
H	-2.72650	4.10588	0.75814
H	4.75908	3.73459	0.91829
H	4.75873	5.32630	1.08342
H	3.37580	4.53918	0.91439
H	1.62491	-4.74808	1.21551
H	-2.50884	-2.94838	1.18746
H	-4.16200	3.71062	1.34505
H	-4.60334	-4.36962	1.57120
H	-3.56687	5.17340	1.60376
H	2.24175	-2.50881	1.35860
H	-6.75438	-2.15872	1.75207
H	6.76404	2.22942	1.79252
H	1.81928	2.39509	1.57653
H	0.19396	3.86896	1.96057
H	7.15765	-1.15216	2.32911
H	-1.99916	-4.19754	2.04811
H	7.66966	1.16825	2.58534
H	6.23754	-4.46148	2.66737
H	0.67789	-3.99065	2.25991
H	1.52202	4.64670	2.39915
H	4.52926	-6.13607	2.92914
H	-2.39644	-0.27636	2.42416
H	-1.60937	-2.70934	2.48935
H	-4.31342	1.32189	2.59442
H	-4.12194	-5.19766	2.85320
H	-5.37010	-4.20232	2.96594
H	1.85084	-4.94311	2.78770
H	-6.89716	-0.07038	3.18744
H	5.71436	5.59807	3.37764
H	3.49307	5.00004	3.16924
H	-0.09924	1.50769	2.84213
H	6.15325	4.05915	3.35084
H	1.18636	0.55447	2.83927
H	-2.86435	1.84634	3.02569
H	3.02310	-1.47255	3.25154
H	5.69128	0.16829	3.52259
H	0.56027	4.01533	3.51153
H	1.50397	-1.94981	3.41111
H	-6.39720	-2.32889	3.84879
H	5.80673	-2.31897	3.91877
H	5.76214	1.71813	3.92419
H	5.34302	-5.89011	4.28501
H	-3.26996	-3.33577	3.88238

H	3.88135	-5.26433	4.10452
H	2.68049	-2.77717	4.11255
H	-3.92232	1.40866	4.14365
H	-7.54086	-0.53017	4.57855
H	5.28517	4.62264	4.57160
H	0.85502	1.44711	4.12563
H	-6.24344	0.40657	4.56809
H	6.38087	-3.54223	4.77602
H	-1.83651	-1.35192	4.42183
H	-1.53845	0.21790	4.51054
H	4.82158	-3.18645	4.83437
H	4.44459	2.70058	5.38163
H	-2.83406	-0.39209	5.22476
H	3.85125	0.38189	5.32587
H	-4.46092	-2.39222	5.49846
H	2.78857	4.42102	5.51687
H	2.29507	0.74299	5.41690
H	1.65857	3.30188	5.69392
H	-5.97329	-2.44132	6.01958
H	-5.16042	-1.06316	6.05106
H	3.25684	1.02784	6.66374
H	2.78601	3.48477	6.81479
N	-4.33554	-1.31472	-2.53602
N	5.00940	1.19627	-1.24555
N	-6.63406	-0.31618	-1.00361
N	-5.04496	2.06623	-0.99393
N	6.63389	0.31628	1.00373
N	5.04495	-2.06624	0.99393
N	-5.00930	-1.19625	1.24566
N	4.33568	1.31458	2.53590
O	-1.97021	0.05311	-0.17774
O	1.97020	-0.05312	0.17774
Rb	0.01396	1.76603	-0.29429
Rb	-0.01398	-1.76605	0.29429
Si	-3.46178	-2.62036	-3.28344
Si	4.39088	1.51217	-2.82786
Si	-4.34538	3.52572	-1.60098
Si	4.34537	-3.52573	1.60098
Si	-4.39089	-1.51218	2.82786
Si	3.46177	2.62034	3.28344
Th	-3.95483	-0.04584	-0.57062
Th	3.95482	0.04583	0.57062

Energy: −1227.92159599 eV

References

1. B. M. Gardner, P. A. Cleaves, C. E. Kefalidis, J. Fang, L. Maron, W. Lewis, A. J. Blake, S. T. Liddle, *Chem. Sci.* **2014**, *5*, 2489.
2. F. F. Puschmann, D. Stein, D. Heift, C. Hendriksen, Z. A. Gal, H. -F. Grützmacher, H. Grützmacher, *Angew. Chem. Int. Ed.* **2011**, *50*, 8420.
3. A. R. Jupp, J. M. Goicoechea, *Angew. Chem. Int. Ed.* **2013**, *52*, 10064.
4. D. E. Bergbreiter, J. M. Killough, *J. Am. Chem. Soc.* **1978**, *100*, 2126.
5. G. M. Sheldrick, *Acta Cryst. Sect. A* **2015**, *A71*, 3.
6. CrysAlisPRO version 39.46, Oxford Diffraction /Agilent Technologies UK Ltd, Yarnton, England.
7. G. M. Sheldrick, *Acta Cryst. Sect. C* **2015***, C71*, 3.
8. O. V. Dolomanov, L. J. Bourhis, R. J. Gildea, J. A. K. Howard, H. Puschmann, *J. Appl. Cryst.* **2009**, *42*, 339.
9. L. J. Farugia, *J. Appl. Cryst.* **2012**, *45*, 849.
10. Persistence of Vision (TM) Raytracer, Persistence of Vision Pty. Ltd., Williamstown, Victoria, Australia.
11. J. Du, C. Alvarez-Lamsfus, E. P. Wildman, A. J. Wooles, L. Maron, S. T. Liddle. *Nat. Commun.* **2019**, *10*, 4203.
12. C. Fonseca Guerra, J. G. Snijders, G. Te Velde, E. J. Baerends, *Theor. Chem. Acc*. **1998**, *99*, 391.
13. G. Te Velde, F. M. Bickelhaupt, S. J. van Gisbergen, A. C. Fonseca Guerra, E. J. Baerends, J. G. Snijders, T. Ziegler, *J. Comput. Chem*. **2001**, 22, 931.
14. E. Van Lenthe, E. J. Baerends, J. G. Snijders, *J. Chem. Phys.* **1993**, *99*, 4597.
15. E. Van Lenthe, E. J. Baerends, J. G. Snijders, *J. Chem. Phys.* **1994**, *101*, 9783.
16. E. Van Lenthe, A. E. Ehlers, E. J. Baerends, *J. Chem. Phys.* **1999**, *110*, 8943.

17. S. H. Vosko, L. Wilk, M. Nusair, *Can. J. Phys*. **1980**, *58*, 1200.

18. A. D. Becke, *Phys. Rev. A*. **1988**, *38*, 3098.

19. J. P. Perdew, *Phys. Rev. B*. **1986**, *33*, 8822.

20. S. Portmann, H. P. Luthi, *Chimia*, **2000**, *54*, 766.
